# An increase in marine heatwaves without significant changes in surface ocean temperature variability

**DOI:** 10.1038/s41467-022-34934-x

**Published:** 2022-12-01

**Authors:** Tongtong Xu, Matthew Newman, Antonietta Capotondi, Samantha Stevenson, Emanuele Di Lorenzo, Michael A. Alexander

**Affiliations:** 1 NOAA Physical Sciences Laboratory, Boulder, CO USA; 2 Cooperative Institute for Research in Environmental Sciences, University of Colorado, Boulder, CO USA; 3 Bren School of Environmental Science and Management, University of California, Santa Barbara, CA USA; 4 Department of Earth, Environmental, and Planetary Sciences, Brown University, Providence, RI USA

**Keywords:** Attribution, Climate and Earth system modelling, Physical oceanography

## Abstract

Marine heatwaves (MHWs)—extremely warm, persistent sea surface temperature (SST) anomalies causing substantial ecological and economic consequences—have increased worldwide in recent decades. Concurrent increases in global temperatures suggest that climate change impacted MHW occurrences, beyond random changes arising from natural internal variability. Moreover, the long-term SST warming trend was not constant but instead had more rapid warming in recent decades. Here we show that this nonlinear trend can—on its own—appear to increase SST variance and hence MHW frequency. Using a Linear Inverse Model to separate climate change contributions to SST means and internal variability, both in observations and CMIP6 historical simulations, we find that most MHW increases resulted from regional mean climate trends that alone increased the probability of SSTs exceeding a MHW threshold. Our results suggest the need to carefully attribute global warming-induced changes in climate extremes, which may not always reflect underlying changes in variability.

## Abstract (web summary)

Increases in high-impact marine heatwaves over the past few decades are found to be due to recent acceleration in long-term ocean surface warming, stressing the need of careful attribution of climate change impact on extreme events.

## Introduction

**Fig. 1:**
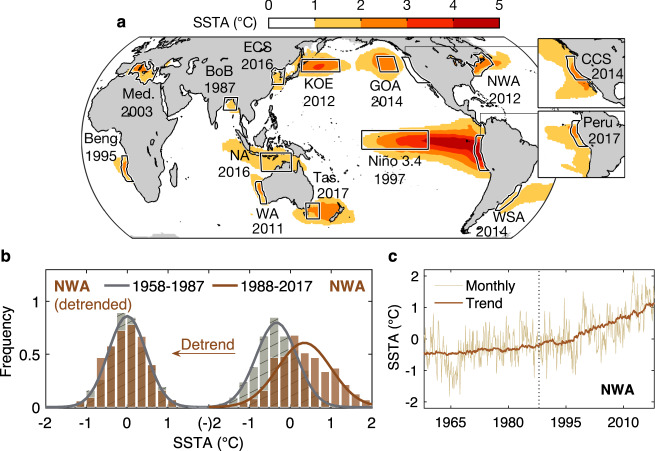
Historical Marine Heatwave (MHW) events and example of nonlinear trend influencing Probability Distribution Function (PDF). **a** Sea Surface Temperature Anomaly (SSTA) of historical MHW events during their peak months, following previous studies^3–5^ and references therein. Outlines mark regions of interest for this study, and the year in which the historical event occurred is also indicated. Peak months of these events are listed in Supplementary. *Beng.* Benguela, *BoB* Bay of Bengal^50^, *CCS* California Current System, *ECS* East China Sea^51,52^, *GOA* Gulf of Alaska, *KOE* Kuroshio-Oyashio Extension^53,54^, *Med.* Mediterranean, *NA* Northern Australia, *NWA* Northwest Atlantic, *Tas.* Tasman Sea, *WA* Western Australia, *WSA* Western South Atlantic. Insets show historical MHWs in the CCS and along the coast of Peru. **b** Histograms (bars) of NWA SSTA during 1958–1987 (gray) and during 1988-2017 (orange), before (right of **b**) and after detrending (left). Lines are PDFs from Gaussian distribution. **c** SSTA time series (thin line) spatially averaged within NWA and the overlaid regional trend (thick line) identified from trend mode (see Methods). Dotted line separates 1958–1987 from 1988 to 2017.

Marine heatwaves (MHWs) are events characterized by prolonged anomalously warm ocean surface or subsurface temperatures^1,2^, seen in many regions of the world’s oceans^3–5^ as shown in Fig. 1a. Over the past several decades, these events have appeared to occur more often and become more extreme and longer-lasting^6,7^, concurrent with long-term global warming^7–9^. The severe ecological and economic consequences of MHWs, including widespread mortality of marine species, adaptive reconfiguration of species ranges, and decline of farmed aquaculture production in commercial fisheries^5,10^, have motivated numerous studies aimed at understanding their drivers and occurrences. Under current warming projections^8,9^, future MHWs are likely to occur at an accelerated rate^11,12^, creating increased urgency regarding the attribution of climate change impacts.

Quantifying the impact of climate change on MHW characteristics relies on identifying the long-term externally-forced warming trend separately from the changes in internal variability (e.g., ref. 13–16). An increase in internal variability of sea surface temperature (SST) acts to widen the probability distribution function (PDF) of ocean temperatures, making extreme warm temperatures more likely. In addition, mean changes due to the warming trend shift the center of the PDF to higher values, also resulting in increased MHW occurrences. However, the long-term SST warming has been observed to occur at a nonlinear rate in the past few decades^17,18^, which is particularly evident in some specific oceanic regions such as the western boundary currents^19^. One example is the Northwest Atlantic (NWA; Fig. 1c), where a slow mean warming was replaced by an accelerated trend in the late 1980s. Comparing PDFs of the consecutive 1958–1987 and 1988–2017 periods (right panel of Fig. 1b, derived from thin line of Fig. 1c) shows not only a positive mean shift but also a broadening of the distribution, suggesting an increase in variance during the later period. However, if we remove the nonlinear trend (i.e., thick line of Fig. 1c) from the NWA index (thin line), the PDFs of the detrended data have almost identical mean and variance over the two periods (left panel of Fig. 1b). That is, the changing trend acts to make the distribution wider, resulting in an apparent increase in variability that can lead to an actual increase of MHW occurrences, despite little change in the natural internal variability itself. This example highlights the importance of carefully distinguishing the long-term warming trend from the internal variability, to avoid conflation of the two on the climate change attribution to MHWs^20^. While attempts have been made to determine the relative role of trend and internal variability in the increasing frequency of MHWs^16^ under continued anthropogenic forcing, no study to date has assessed the potentially important additional role of the trend’s nonlinearity.

In this paper, we conduct a global analysis of MHW events over the past 60 years that separates the long-term trend, including its nonlinear change over time, from the internal ocean variability, thereby allowing for better estimates of the impact of climate change on both MHWs and overall SST variability. We note, however, that the nonlinearity of the trend shown in Fig. 1c primarily stems from the acceleration of the trend starting in the late 1980s. This period approximately coincides with the advent of the satellite era, which led to a dramatic increase in the density of SST observations^21^, and could potentially result in an apparent change of the magnitude of the trend. To ascertain that the nonlinear trend we discuss in this study is not an artifact of the changes in the observing techniques, we repeat a similar analysis on the Coupled Model Intercomparison Project phase 6^22^ (CMIP6; see Methods) multi-model ensemble, focusing on the same historical period covered by the observations. As we will see, the similarity of the climate models’ results suggests that the nonlinear character of the trend is a real climate signal and not a result of the changes in observational sampling. In addition, to increase the sample size, we also use a long integration of a linear inverse model^23,24^ (LIM; see Methods). Both CMIP6 and LIM simulations provide long records of “alternative histories” of what could have happened, allowing a more robust assessment of the significance of our results. The LIM, a multivariate empirical dynamical model, represents spatially-varying climate anomalies as a combination of linearly deterministic (i.e., predictable) dynamics plus an unpredictable nonlinear residual approximated by white noise^23,24^. The LIM has been extensively used in predicting seasonal-to-interannual surface ocean conditions (e.g., ref. 25–27), but it has also been run as a climate simulation model. In this case, the LIM can generate climate realizations whose spatiotemporal evolution is statistically consistent with past observations, which has allowed the LIM’s use for testing hypotheses including whether El Niño has significantly changed over the last several decades^28^, the extent to which tropical Pacific decadal variability is a residual of El Niño events^29^, and the impact of tropical and extratropical processes upon evolving Northeast Pacific MHW events^30^. Motivated by these earlier results, we generate large LIM ensembles that realistically represent the dynamics of anomalous SST evolution over 1958–2017, allowing us to identify large samples of simulated MHWs and to diagnose the impact of climate change on SSTs, analogous to our use of CMIP6 realizations. Using both LIM and CMIP6 ensembles, we provide a comprehensive analysis of MHW changes in both ensembles and how those compare to the observed changes.

## Results

### Mean change of sea surface temperature and the observed nonlinear trend

**Fig. 2:**
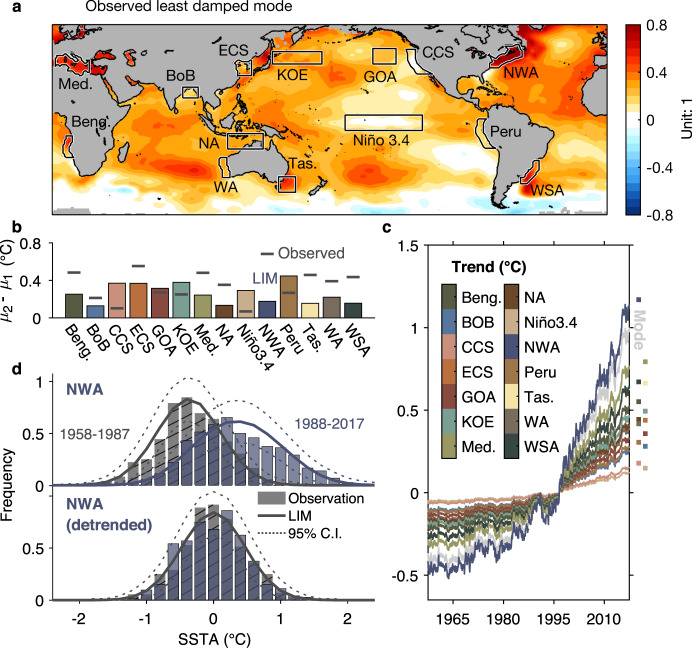
Sea Surface Temperature (SST) trends over the historical period. **a** Pattern of the trend mode (Unit: dimensionless) over 1958–2017 (see Methods). **b** Regional mean changes, calculated as differences between mean SST Anomaly (SSTA) during 1958–1987 (${\mu }_{1}$) and 1988–2017 (${\mu }_{2}$) at each targeted region. Horizontal lines are observed mean changes. Bars mark the edges of the 95% confidence interval of mean changes, obtained from a 3000-member LIM ensemble. The LIM was constructed from detrended observations and reanalyses over the 1958–2017 period (the “LIM5817” ensemble; see Methods). **c** Regional trend time series, determined from the pattern (**a**) and the time series (gray line) of the trend mode. **d** Probability Distribution Functions (PDFs) of Northwest Atlantic (NWA) SSTA during 1958–1987 (gray) and 1988–2017 (blue), before (top of **d**) and after detrending (bottom). Bars show the observed SSTA histograms (same as Fig. 1b). Solid curves represent the ensemble mean PDFs determined from the LIM5817 ensemble (bottom) and from the trend+LIM5817 ensemble (top), respectively. The dotted lines represent the 95% confidence intervals.

We begin by identifying the pattern and amplitude of the observed long-term trend and estimating where it is statistically significant. We use the least damped eigenmode of the LIM’s dynamical operator to extract the trend component (see Methods), a technique established by several previous studies^13,31,32^. The resulting trend pattern (Fig. 2a) appears generally robust since we found a similar pattern using other approaches, including computing the “mean shift” as the difference between the 1958–1987 and 1988–2017 means (Fig. 2b and Supplementary Fig. 1) or fitting a piecewise linear trend at each grid point (see supplementary information), and other studies have also obtained similar results^15,33,34^. The time series associated with the trend pattern (gray line in Fig. 2c) shows an acceleration during the second half of the observational period, which is also seen in the evolution of regionally averaged SST anomalies (Fig. 2c). However, in some regions like the Niño 3.4 domain and the California current system, the trend identified from the least damped eigenmode is very weak (Fig. 2c and Supplementary Fig. 1), consistent with the overall SST trend in those regions being linked to residual El Niño-Southern Oscillation (ENSO) variability^35^.

We test the statistical significance of the observed local trends against a large set of climate realizations (or alternative histories) based on the LIM’s realistic representation of the observed dynamical system^27^. First, we remove the least-damped eigenmode from the LIM so that it represents “detrended” dynamics. Then this LIM is used to generate a 3000-member ensemble of 60-year-long realizations which overall share the same spatial and temporal covariability statistics as the observations during 1958–2017. For each ensemble member, however, while SSTA variations are governed by the same deterministic dynamics, representing predictable anomaly evolution, they are driven by different random noise realizations, representing unpredictable internal variability. This simulation is hereafter denoted LIM5817; see Methods. For each region of interest, a mean shift in observations is then considered 95% significant only if it falls outside the 2.5–97.5% range of the 3000 mean shifts (shown by the bars in Fig. 2b) drawn from the LIM5817 ensemble. By this criterion, Fig. 2b shows that the regions of Benguela, Bay of Bengal, East China Sea, Mediterranean, Northern Australia, Northwest Atlantic, Tasman Sea, Western Australia and Western South Atlantic all experienced significant mean shifts (see also Supplementary Fig. 1).

### Impact of the observed nonlinear trend on variance

As noted in the introduction, the accelerated historical trend in the Northwest Atlantic (Fig. 1c) has led to an apparent variance increase (Fig. 1b). This effect is largely removed after nonlinear detrending (Fig. 1b). We can use the LIM ensembles to better understand these results.

First, note that the PDFs determined from the LIM5817 ensemble are a good fit to the (independently constructed) histograms of detrended SSTA over both 1958–1987 and 1988–2017 (solid and dotted lines in bottom panel of Fig. 2d), indicating that the two periods are statistically indistinguishable when the trend is removed. Next, the observed trend component is added back to each LIM5817 ensemble member, so that the resulting “trend+LIM5817” ensemble now represents both the trend and the variability during 1958–2017. PDFs are then determined from the trend+LIM5817 ensembles, separately for the first half (i.e., 1958–1987) and the second half (i.e., 1988–2017) of the 60-yr period. By construction, any difference between these two simulated PDFs is only due to the externally forced trend, since the dynamical system driving the underlying internal variability is unchanged (i.e., it is generated by a single LIM, whose ensemble-mean distribution is shown by the solid line in the bottom panel of Fig. 2d). The top panel of Fig. 2d shows that the simulated PDFs of the two periods match the observed PDFs, notably capturing the change between the two periods. This suggests that the accelerating trend was responsible not only for the overall shift to warmer values but also for the widening of the PDF. A similar result is seen in other regions with strong nonlinear trends (e.g., the Mediterranean region, Fig. 2c; see also Supplementary Fig. 2), while the effect is weaker in regions with weaker trends (see Supplementary Fig. 2 for other regions).

### The CMIP6 nonlinear trend and its effect on variance

**Fig. 3:**
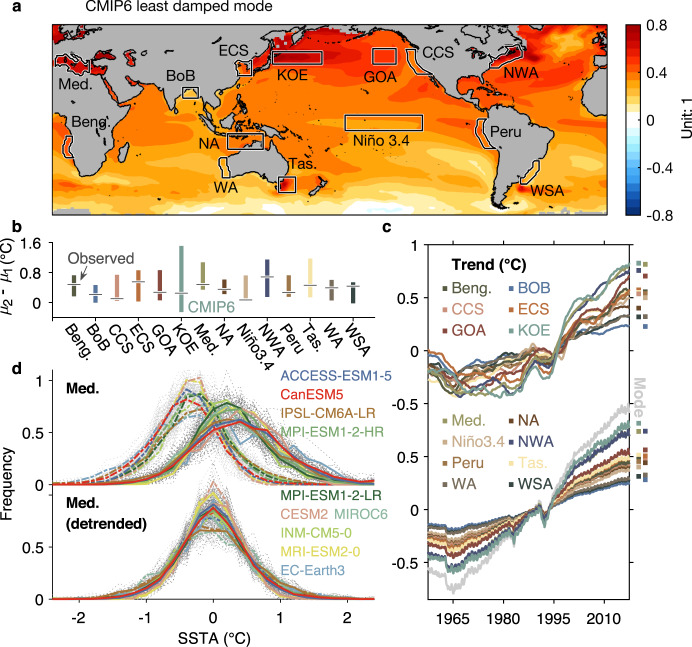
CMIP6 Sea Surface Temperature (SST) trends over the historical period. **a** Pattern of the CMIP6 trend mode (Unit: dimensionless) over 1958–2017. **b** Regional mean changes derived from CMIP6 (bars), compared with observed changes (horizontal lines; same as Fig. 2b). Bars are 95% confidence interval of mean changes from CMIP6 realizations. **c** Regional trend time series, (top) determined by applying 60-month moving average to the CMIP6 ensemble mean time series, vs. (bottom) determined by computing the trend mode for the CMIP6 models. **d** Probability distribution functions (PDFs) of Mediterranean SST Anomaly (SSTA) during 1958–1987 (dashed lines) and during 1988–2017 (solid lines), before (top) and after (bottom) detrending. Mean PDFs of each CMIP6 model are separately plotted in a different color. Light (dark) gray dotted lines are PDFs of each CMIP6 realization during 1958–1987 (1988–2017).

To assess how well the observed trend and its impact on the distributions are reproduced by the CMIP6 historical simulations, we repeat the analysis of Fig. 2 on the output of 10 models (in total 133 realizations; see Methods) from the CMIP6 set of ensembles (Fig. 3). Similar to our approach in the observational analysis, we first derive the trend from the least damped eigenmode of LIMs separately constructed from each CMIP6 realization of each model (Supplementary Fig. 3). The ensemble mean of these trend patterns, in which each model’s average is first calculated prior to computing the multi-model ensemble mean, is shown in Fig. 3a. As we found for observations, the LIM-based trend pattern reproduces the trend pattern determined instead by using the mean shift between the two periods of the CMIP6 realizations (Supplementary Fig. 4 and bars of Fig. 3b), again demonstrating that the least damped mode can robustly identify the trend. The time series associated with the trend pattern (gray line in lower portion of Fig. 3c) and its expression in the different regions of interest (lower portion of Fig. 3c) exhibit a nonlinear character as seen in observations (Fig. 2c). These time series derived from the LIM’s least damped eigenmode are also consistent with those directly obtained from the ensemble average of the CMIP6 models (upper portion of Fig. 3c), which is expected to yield the long-term forcing signal by averaging out the internal variations of individual ensemble members. Notably, the regional trends identified from the least damped mode (lower portion of Fig. 3c) sharply capture the sudden temperature decrease around 1991, which is likely associated with the Mount Pinatubo eruption and which is also present, albeit less well-defined, in the CMIP6 simulations^22,36^. These results further indicate the robustness of using the LIM’s least damped mode to identify the externally forced signal, as well as supporting the hypothesis that the long-term trends over the historical period have been evolving nonlinearly.

The long-term trends simulated in the CMIP6 ensemble are, overall, similar to the observed trend. However, it is worth noting that large regions of the North Pacific, especially the Kuroshio-Oyashio Extension, warmed more substantially over the historical period in the CMIP6 ensemble-mean (Fig. 3a) than was observed (Fig. 2a). Similarly, the Nino3.4 region in the tropical Pacific displays a faster warming in the CMIP6 simulations compared to observations^37^ (Fig. 3b). These issues may suggest a systematic bias of CMIP6 models^38^, which is beyond the scope of this study.

To examine the influence of the nonlinear trend upon the CMIP6 model SST distributions, we choose the Mediterranean region, which has experienced significant warming both in observations (Fig. 2b, c) and in the CMIP6 simulations (Fig. 3c). Figure 3d shows the resulting PDFs derived from each CMIP6 realization (dotted lines) and the averaged PDFs for each CMIP6 model (dashed and solid lines). As in observations (Fig. 2d), the accelerating trend in the CMIP6 simulations leads to both a mean shift and a widening of the distribution. For other regions with smaller simulated trends, the impact on the distribution is correspondingly weaker (not shown). This result also suggests that where the CMIP6 models overestimate the trends, they therefore may also overestimate changes to the distributions.

### Modest changes in internal variability of sea surface temperature

**Fig. 4:**
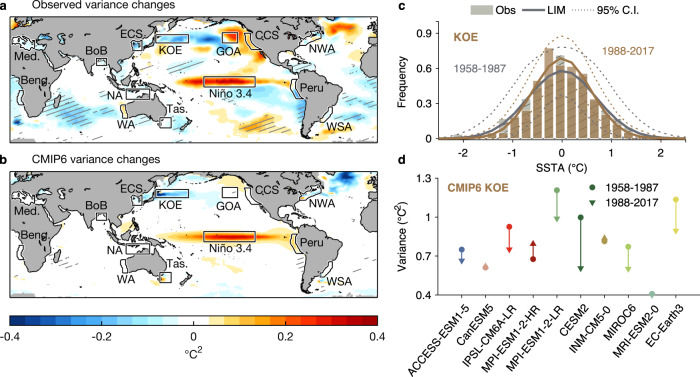
Changes of detrended Sea Surface Temperature (SST) variance over the historical period. **a** Observed and **b** CMIP6-simulated variance changes from 1958–1987 to 1988–2017, in units of (°C)^2^. Hatched regions indicate locations where the observed variance changes are significant, i.e., outside the 95% range of variance changes derived from **a** the LIM5817 ensemble, and **b** CMIP6. **c** Probability Distribution Functions (PDFs) of detrended SST Anomaly (SSTA) during 1958–1987 (gray) and 1988–2017 (orange) in the Kuroshio-Oyashio Extension (KOE) region. Bars show the histogram of observed detrended SSTA. Solid curves and dotted lines are the ensemble mean PDFs and 95% confidence interval, respectively, obtained from LIMs constructed over the two periods separately (“LIM5887” and “LIM8817”; see Methods). **d** Variance of detrended SSTA during 1958–1987 (circles) and during 1988–2017 (triangles) derived from each CMIP6 model in the KOE region. Upward (downward) triangles represent variance has increased (decreased) between the two periods.

While the intensifying trend has been shown to contribute to an apparent increase in variance, it remains possible that the underlying internal variance could also be changing under anthropogenic forcing, at least in some regions. To assess such potential changes, we first determine the “detrended variance” from the observed detrended SSTA. The resulting difference map between the detrended variance for the two periods 1958–1987 and 1988–2017 (Fig. 4a) shows notable increases in the detrended variance within the Niño 3.4 and the Northeast Pacific regions and decreases in the Kuroshio-Oyashio Extension, East China Sea and large regions of the Southern Oceans. Such variance changes are also reflected in the SSTA distributions, such as a narrowing of the PDFs from 1958–1987 to 1988–2017 in the Kuroshio-Oyashio Extension region (histogram bars in Fig. 4c). The increase in variance in the Northeast Pacific is consistent with the increase in variance in the central equatorial Pacific seen in Fig. 4a, as Northeast Pacific MHWs appear to be associated with the occurrence of Central Pacific El Niño events^39,40^. However, the variance decrease in the KOE region remains difficult to explain.

Next, we assess the significance of the observed changes in detrended variance, by asking whether they could have occurred by chance within a statistically stationary system. This question is addressed by comparing these observed changes against the range of potential variance changes from the first to second halves of each realization of the LIM5817 ensemble (see Methods); 95% significance levels are indicated by hatching in Fig. 4a. Interestingly, in those regions where high-impact MHWs have occurred (e.g., outlines in Fig. 4a), changes in the detrended variance are not significant. That is, these observed variance changes are small enough that they could have occurred because of random changes in internal variability, without being associated with a systematic change in variability related to the external forcing. For example, we know that ENSO variability in the equatorial Pacific undergoes naturally occurring decadal variations (e.g., ref. 41). Thus, it is not surprising that the changes in variance in the tropical Pacific are within the range of internal variability and cannot be attributed to climate change.

Despite the lack of significance in the variance changes, however, many aspects of the observed pattern of detrended variance changes are reproduced by the CMIP6 historical simulations, or at least by the multi-model ensemble mean. We repeat the above analysis for all the CMIP6 model ensembles, finding the changes in the detrended variance between period 1958–1987 and 1988–2017 for each CMIP6 realization (Supplementary Fig. 5). The CMIP6 ensemble mean result (Fig. 4b) shares some notable features with the observational map in Fig. 4a, including the detrended variance increase within the Niño 3.4 region and the decrease in the Kuroshio-Oyashio extension region. The CMIP6 models also indicate a detrended variance increase in the northeast Pacific, although much weaker than observed. On the other hand, the CMIP6 ensemble-mean variance changes differ from the observations in the North Atlantic and in much of the Southern Oceans.

These differences may be due to large model spread and low model resolution of CMIP6. Indeed, several models do not reproduce the overall observed detrended variance changes. This is illustrated for the Kuroshio-Oyashio extension region in Fig. 4d. The majority of the CMIP6 models simulate a decrease in the detrended variance: 6 out of 10 models have decreases, 3 models have almost no changes, and 1 model shows an increase. These inter-model differences as well as those found in other regions (Supplementary Fig. 5) indicate the necessity of using many realizations to improve the modeled representation of the observed changes, although this does not rule out model disagreement. These biases between models and observation may be linked to the underestimated upper-ocean heat flux convergence due to the low model resolution and hence the lack of resolving mesoscale ocean eddies (e.g., ref. 42, 43).

### Quantifying the impact of climate change on marine heatwaves in observations and models

**Fig. 5:**
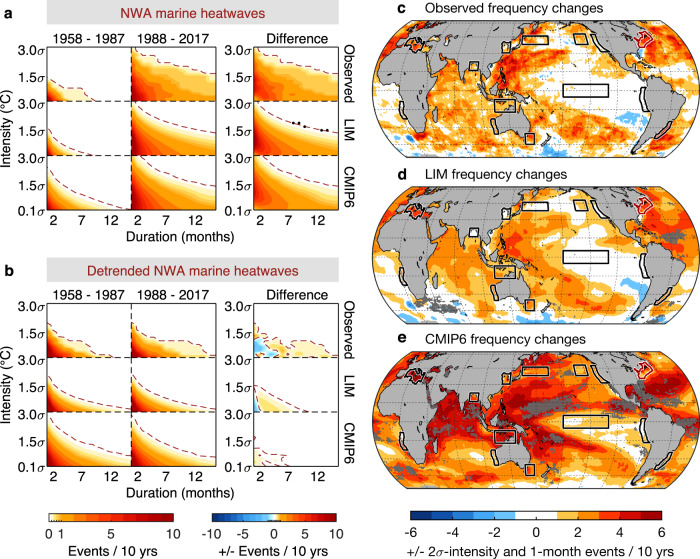
Impact of changes in the trend on Marine Heatwave (MHW) occurrences. **a** Northwest Atlantic (NWA) MHW frequencies (events per decade) over a range of intensities and duration, i.e., Intensity-Duration-Frequency (IDF) plot, during 1958–1987 (first column), 1988–2017 (second column) and their difference (third column). First/second/third row is derived from observed Sea Surface Temperature Anomaly (SSTA)/trend+(LIM5887 ensemble, LIM8817 ensemble)/CMIP6 (see Methods). Dashed lines represent the contour level of 0.1 events (red) or −0.1 events (blue) per decade (i.e., ±1 event per century). Black dots in the third column mark observed values that are 95% significantly different from the climate ensemble (see Methods). **b** Same as panel a except that MHWs are derived from detrended dataset: observed detrended SSTA (first row), LIM5887 and LIM8817 (second row), and detrended CMIP6 (third row). **c**–**e** Changes in the frequencies of **c** observed, **d** trend+(LIM5887, LIM8817), and **e** CMIP6 MHW events, between 1958–1987 and 1988–2017. In these spatial plots, an MHW event is identified as SSTA reaching an amplitude (intensity) of at least 2$\sigma$ and thereafter persisting for at least 1 month (duration), following a previous study^1^. Positive (negative) represents increase (decrease) of MHW occurrences per decade. Gray dots in **d**, **e** mark where the observed values are 95% significantly different from the climate ensemble.

MHWs may be quantified using measures of their intensity and duration^30,44^, and then the effects of climate change on MHWs may be assessed by changes in these measures. For example, in the Northwest Atlantic (top row of Fig. 5a), MHW occurrences during the years 1958–1987 and 1988–2017 are quantified by determining how often events reaching given intensity and duration thresholds occurred in each period, with the results displayed as intensity-duration-frequency (IDF) plots (see ref. 44 and Methods). The frequency of MHW events for any given threshold value notably increased between the two periods, i.e., events that rarely occurred during 1958–1987 occurred substantially more often during 1988–2017.

To examine the global extent of the increases in MHW occurrences, we repeat the analysis of Fig. 5a at each ocean grid location, i.e., we compute IDFs at each grid point. To condense this analysis into a single figure, we show results using one representative definition of MHW events, those that reach at least 2$\sigma$ intensity and persist for at least 1-month duration (Fig. 5c). Using this representative definition, we find that the observed MHW frequencies have increased almost worldwide, especially in the Western Pacific warm pool, Indian Ocean, and Atlantic.

We next evaluate how well these observed MHW increases are reproduced in the large climate ensemble of LIM. Recall that, by construction, within our trend+LIM5817 ensemble any statistically significant change in MHWs is due only to the externally-forced trend. To capture changes in MHW statistics induced by significant changes in the internal variability (i.e., due to changes in the dynamical system), we construct two distinct LIMs—one from the detrended anomalies of each 30-year subperiod (referred to as LIM5887 and LIM8817; see Methods)—and then use each of them to generate 3000-member 30-year ensembles for each period. We then add the observed trend component during 1958–1987 (1988–2017) back to each member of the LIM5887 (LIM8817) ensemble. The resulting “trend + (LIM5887, LIM8817)” ensemble allows us to assess the influence of climate change, combining changes in the internal dynamics controlling the variability (e.g., solid and dotted lines in Fig. 4c) and the externally-forced trend, on MHW statistics. For example, we find that the trend + (LIM5887, LIM8817) ensemble can simulate the increased occurrences of extreme Northwest Atlantic MHW events for a wide range of intensities and durations (second row of Fig. 5a), similar to the observed changes (first row) with 95% statistical significance. The global extent of the observed MHW increases (Fig. 5c) is also reproduced in this LIM ensemble (Fig. 5d) with 95% statistical significance (only 2% of the ocean area show the observed values outside 95% ranges of the LIM ensemble).

Similarly, the CMIP6 ensemble can also reproduce the overall MHW increases, both in the Northwest Atlantic region (third row of Fig. 5a) and in large regions of the global ocean (Fig. 5e). Note that the overwarmed CMIP6 ensemble (Fig. 3a) also appear to overestimate the changes in MHW events, significantly so in the tropical regions (gray dots of Fig. 5e). Choosing other regions or other pairs of thresholds, to examine changes in observed MHWs and evaluate how those are reproduced by the LIM and the CMIP6 ensemble, yields a qualitatively similar picture (Supplementary Figs. 6–7).

### Separating the effects of changes in trend and variability upon marine heatwave frequency

**Fig. 6:**
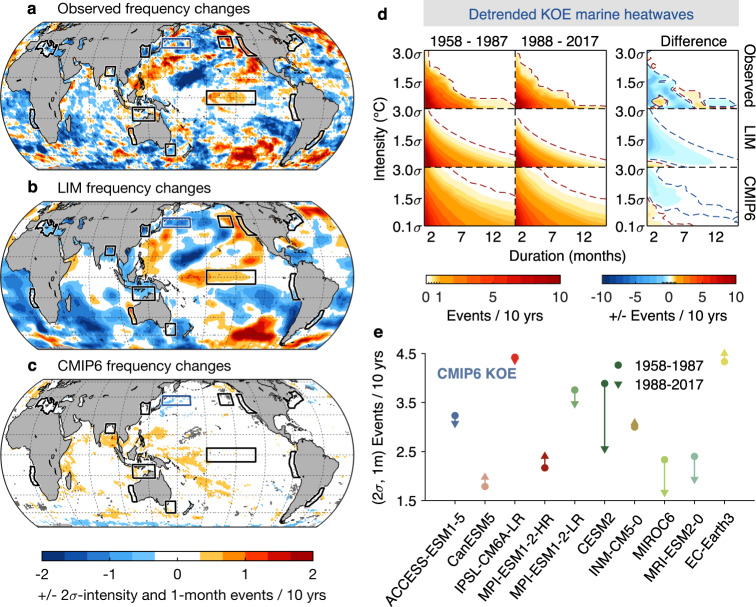
Impact of changes in the internal (detrended) variability on Marine Heatwave (MHW) occurrences. **a**–**c** Same as Fig. 5c–e except that MHWs are derived from detrended dataset: **a** observed detrended Sea Surface Temperature Anomaly (SSTA), **b** LIM5887 and LIM8817, and **c** detrended CMIP6. **d** Same as Fig. 5b except for Kuroshio-Oyashio Extension (KOE). **e** MHW frequencies during 1958–1987 (circles) and during 1988–2017 (triangles) derived from each detrended CMIP6 model at the KOE region. Note that, in all panels except d, MHW events are identified based on a 2$\sigma$ intensity and 1 month duration.

If we now remove the long-term trend and examine the frequencies of MHWs from the detrended time series, for instance for the Northwest Atlantic region, we find that the large increases of MHW occurrences seen in the last column of Fig. 5a have been reduced to only minor changes after the trend is removed (last column of Fig. 5b). This is evident in the observed record, the LIM ensemble and the CMIP6 models. This is also seen globally. That is, the global increase in MHWs (Fig. 5a–c) is considerably reduced after removing the long-term trend (Fig. 6a–c), and in some regions MHW occurrences even decrease (e.g., Fig. 6d). The comparison of MHW statistics before and after detrending suggests that the long-term trend is the major contributor to the increase of MHW occurrences in both observations, LIM and CMIP6 models and may on its own explain most, if not all, global changes in MHW occurrences.

The trend similarly impacts the frequency of MHW events for the other regions examined in this study, with the intensity of this effect related to the strength of the warming (Supplementary Fig. 6). We also find that if we had only considered detrended variability, changes in the MHW occurrences would be spatially heterogenous, with increasing (decreasing) variance leading to increasing (decreasing) MHW frequencies (cf. Figs. 4a with 6a). These MHW changes driven solely by changes to the internal variability are generally reproduced by the (LIM5887, LIM8817) ensembles (Fig. 6b), whereas MHWs have changed only slightly in the CMIP6 multi-model ensemble (less than ±1 event per decade; Fig. 6c).

Changes in CMIP6 MHW statistics, although small, might have inter-model differences as was seen for the detrended variance (Fig. 4d). To examine this, we return to the Kuroshio-Oyashio Extension and show in Fig. 6e the changes of MHW occurrence in each CMIP6 model. Results show that the majority of the CMIP6 models simulate a decrease in MHW occurrences –6 out of 10 models have decreases, and 4 models are slightly increasing. These inter-model differences are overall consistent with those shown for the detrended variance (cf. Figs. 4d with 6e), supporting that the changes in the detrended variance are linked to changes in MHW occurrence. This also suggests that an improved model representation through, e.g., increasing model resolution, may improve the capture of observed SST variance and the associated MHW occurrences.

So far, we have analyzed the impact of internal variability on the MHWs identified from representative thresholds, i.e., 2$\sigma$ intensity and 1-month duration (Fig. 6a–c, e). To show that these are a robust representation of the internal variability, we examine the MHW frequencies associated with a range of intensities and durations, derived from the detrended datasets for the KOE region (Fig. 6d). The IDF plots derived from the observed record show overall decreases of MHW occurrences, except for small portions of the IDF diagram. The general decrease of events, as well as the small increase in low intensity events, are reproduced by the (LIM5887, LIM8817) ensembles and CMIP6. Similar changes are also seen in other regions with increases or decreases in MHW frequency generally consistent with the corresponding change in variance (Supplementary Figs. 6–7).

### Marine heatwave changes more pronouncedly induced by trend than by variability

**Fig. 7:**
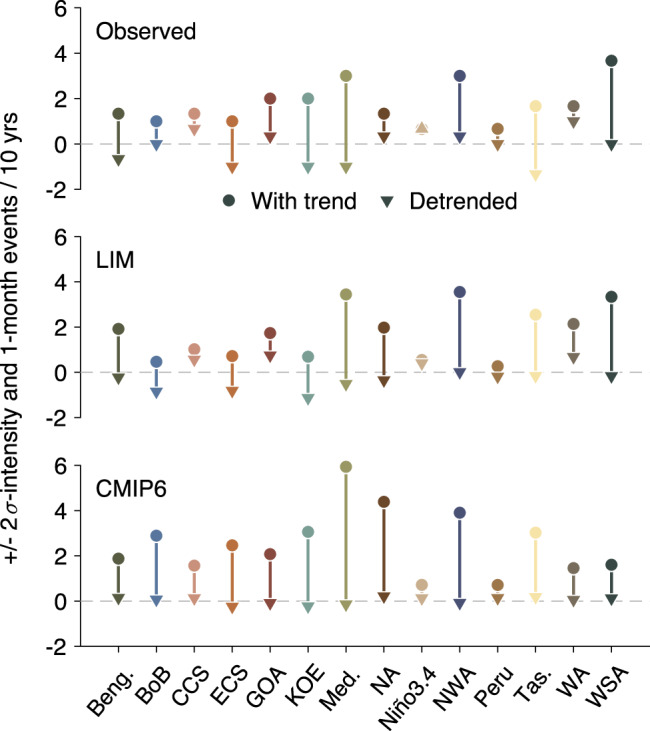
Changes in Marine Heatwave (MHW) occurrences at regions of historical events, before (circles) and after detrending (triangles). Circles (triangles) represent increased or decreased MHW frequencies between 1958–1987 and 1988–2017, derived from dataset with the trend (without trend): (top) observed, (mid) LIM, and (bottom) CMIP6. Note that MHW events are identified based on the 2$\sigma$ intensity and 1 month duration. Qualitatively similar results are found using other pairs of thresholds to define MHW events (Supplementary Fig. 8).

Lastly, we summarize the changes of MHW occurrences induced by the long-term trend as opposed to those induced by changes to the internal variability, in regions where historical MHW events occurred. This is done by quantifying, in each region of interest (as shown in Fig. 1a), the change in MHW occurrence between 1958–1987 and 1988–2017 (circles in Fig. 7) relative to the changes obtained between the two detrended periods (triangles).

From the observed record, we find increases of 2$\sigma$ MHW occurrences in all regions of interest (positive circles in top panel of Fig. 7), with the most pronounced increase seen in the Mediterranean, Northwest Atlantic and Western South Atlantic. These increases are largely reduced after detrending (the downward changes from circles to triangles). That difference (the line segment between a circle and a triangle) thus solely represents the impact of trend on MHW occurrences. For example, the increased 2$\sigma$ MHW occurrences in the Northwest Atlantic (positive circle) can be entirely attributed to the long-term trend (line segment), as the minimal change in internal variability has no effect (triangle near 0).

The impact of trend vs. variability on the observed MHWs is overall reproduced by the LIM ensembles (middle panel of Fig. 7) and the CMIP6, although CMIP6 attributes almost all changes of MHW occurrences to the trend at all regions of interest (bottom panel). Moreover, changes in MHW occurrences induced by changes in internal variability are overall statistically insignificant, as they are not significantly different from 0 (Supplementary Fig. 9). Overall, our results suggest that the long-term trend is responsible for a large portion of the increased MHW events, whereas externally-forced changes to the internal variability is secondary.

## Discussion

Recent research has identified many regions worldwide where MHWs appear to be occurring more frequently and with increased severity. However, in most regions with historical events, the SST variance has either stayed relatively constant or even decreased. Although these increases and decreases in the SST internal variance may eventually be attributed to climate change as longer data records become available, at this time results from the LIM and CMIP6 analyses show that these changes are generally not statistically significant.

Due solely to the influence of these variance changes, the occurrence of MHWs would have changed only slightly, with increasing (decreasing) variance leading to increasing (decreasing) MHW frequencies. Yet instead, we found increasing occurrences of MHWs over a wide range of intensity and duration in almost all the regions of interest. These increases are mainly attributable to the historical trend, which recently accelerated, shifting the mean background climatology to become increasingly warmer at a faster rate in most regions. This intensifying trend has therefore led to both a mean warming effect and even, in some locations, to an *apparent* increase of the variance, both shifting the probability distribution to warmer values and widening it. The impact of the observed warming trend on MHW frequency either amplified or offset, respectively, the regional increase or decrease in SST variance driven by internal variability. The main contribution of the nonlinearly evolving trend is also evident in large ensembles of LIM simulations and CMIP6 multi-model realizations.

Still, there are a few regions where SST variance—even relative to the background trend—has increased over the past 60 years (i.e., in the Northeast and tropical Pacific). By acting to widen the distribution of SSTA, the variance increase in these few regions may have driven increased occurrences of extreme events (e.g., the Northeast Pacific MHW event during 2013–2015), although even in these cases the impact of the nonlinear trend may have mattered more.

Although the historical period has experienced a nonlinearly growing trend and minor changes in internal variability, it remains to be seen whether such changes will continue in the remaining portion of the 21st century. For instance, we might in the future experience an increasing rate of greenhouse gas emissions, causing the nonlinear aspect of the trend as well as its impact on marine extremes to be more pronounced. In addition to the trend, changes in the climate system may alter the variability. In a future projection study^15^, the variability changes were found to be small and spatially heterogenous over most of the global ocean; however, in the high-latitude regions (>60°N) that are seasonally or constantly covered by ice, the melting of sea ice leads to a large increase in SST variability in the future, since SSTs are no longer constrained to remain near the freezing point of sea water. That is, in high-latitude regions, we might expect that both the warming trend as well as the increasing variability play an important role in increasing warm ocean anomalies.

Finally, our findings and analyses highlight the importance of carefully distinguishing between the long-term warming trend and externally forced changes in the dynamical system, and their impacts upon MHWs as well as other climate and marine extremes. Our LIM diagnostic approach provides a clear path for future assessment of separating the relative influence of trend and variability on extreme ocean events.

## Methods

### Sea surface temperature (SST) and sea surface height (SSH) data

Monthly SSTs from the Hadley Centre Sea Ice and Sea Surface Temperature (HadISST)^21^, the Extended Reconstructed Sea Surface Temperature version 5 (ERSSTv5)^45^, and the Centennial in situ Observation-Based Estimates (COBE)^46^, were used in our analysis. These SST products were interpolated onto the same grid as the HadISST dataset (1^o^$\times$1^o^ spatial resolution), and the averaged SST products were analyzed, following a previous study^26^. The monthly SSHs from the European Centre for Medium-Range Weather Forecasts (ECMWF) Ocean Reanalysis System 4 (ORAS4)^47^, were analyzed. The global domain extended from 60°S to 64°N. The temporal range was for the period 1958–2017. The climatological annual cycle computed from the full length of the historical record at each grid point was removed to obtain the SST anomaly (SSTA) and SSH anomaly (SSHA).

While our study focuses on changes during the historical period of 1958–2017, the mid-point of splitting this period into two halves (i.e., 1958–1987 and 1988–2017) coincides approximately with the time when satellites are introduced (around 1980). To assess whether our results are sensitive to data coverage, we examined the variance changes during the post-satellite era, by subtracting the SST variance during 1982–1999 from the SST variance during 2000–2017 (Supplementary Fig. 10). We found that the changes in SST variance during the post-satellite era were overall consistent with the historical period of 1958–2017 (Fig. 4a), suggesting our key results are not merely due to changes in data coverage but reflect real observed changes.

In addition, we examined the large climate ensemble of CMIP6^22^ realizations, which did not assimilate any observations but were controlled by the prescribed external forcing (e.g., greenhouse gases, aerosols) and climate model dynamics. Assessing the CMIP6 runs serves as an independent confirmation that our results are not an artifact of the changes in data coverage associated with the introduction of satellite data.

Note that CMIP6 historical runs end in 2014. To utilize the CMIP6 realizations, we initially retrieved SSTs and SSHs from all CMIP6 models with more than 10 historical runs for the period between 1955 and 2014. Results based on this period (not shown) are qualitatively similar to the period 1958–2017. Still, to analyze the same period as we used for observations, we retrieved the shared socioeconomic pathway scenario 7.0 (SSP3-7.0) future runs, representing the business-as-usual future projections. We then concatenated the historical runs during 1958–2014 with the future runs during 2015–2017, so that we analyzed CMIP6 1958–2017 data. Each CMIP6 realization had its climatological annual cycle separately determined and removed to obtain the SSTA and SSHA. Table 1 lists all models analyzed; note that our analysis is constrained by the available number of future runs, which is less than historical runs.

**Table 1:** Number of historical runs, and future runs of CMIP6 models analyzed in this study

Model	Historical	SSP3-7.0
ACCESS-ESM1-5	40	40
CESM2	11	3
CanESM5	25	25
EC-Earth3	22	2
EC-Earth3-CC	10	0
GISS-E2-1-G	12	0
GISS-E2-1-H	10	0
INM-CM5-0	10	4
IPSL-CM6A-LR	33	11
MIROC6	50	3
MPI-ESM1-2-HR	10	10
MPI-ESM1-2-LR	30	30
MRI-ESM2-0	10	5
NorCPM1	30	0
Total	303	133

### Linear Inverse Model (LIM)

The time evolution of a climate state ${{{{{\bf{x}}}}}}$ may often be approximated by the stochastically forced linear dynamical system,

1 $$\frac{{{{{{\rm{d}}}}}}{{{{{\bf{x}}}}}}}{{{{{{\rm{d}}}}}}t}={{{{{\bf{Lx}}}}}}+{{{{{\boldsymbol{\xi }}}}}}$$

where ${{{{{\bf{x}}}}}}(t)$ is the climate state, ${{{{{\bf{L}}}}}}$ is a linear dynamical operator, ${{{{{\boldsymbol{\xi }}}}}}$ is a vector of temporally white noise that may have spatial structure (determined from a balance condition derived from (1)), and $t$ is time. Determining (1) from observed covariances results in a linear inverse model (LIM^24^). LIMs are typically low-order models, where the state vector is expressed in a reduced empirical orthogonal function (EOF) space. In this paper, for the observed LIMs, ${{{{{\bf{x}}}}}}(t)$ represents the leading principal components (PCs) of observed SSTA (29 PCs) and reanalysis SSHA (22 PCs), where the EOFs for each field were separately determined, explaining 78.6% and 73.7% of each corresponding field’s total variance. The lag-covariance used to determine the LIM operators was computed using a training lag of ${\tau }_{0}=1$ month. We also tested to make sure the results were not sensitive to this choice, as is generally done when constructing a LIM. See^26,27,30,48^ for other details concerning the LIM and its construction.

### Process of identifying the trend from the linear dynamical operator

Several studies^13,31,32^ have shown how the externally forced trend is captured by the least damped eigenmode of ${{{{{\bf{L}}}}}}$. To identify the trend, we performed an eigenanalysis on ${{{{{\bf{L}}}}}}$; that is,

2 $${{{{{\bf{LU}}}}}}={{{{{\bf{U}}}}}}{{{{{\boldsymbol{\Lambda }}}}}}$$

where ${{{{{\bf{U}}}}}}$ is the matrix of eigenvectors and ${{{{{\boldsymbol{\Lambda }}}}}}$ is the diagonal matrix of eigenvalues (${\lambda }_{i}$). ${{{{{\bf{V}}}}}}$, the eigenvectors of ${{{{{\bf{L}}}}}}$’s adjoint, is simply determined by ${{{{{{\bf{V}}}}}}}^{{{{{{\rm{H}}}}}}}{{{{{\boldsymbol{=}}}}}}{{{{{{\bf{U}}}}}}}^{-1}$, such that ${{{{{{\bf{L}}}}}}}^{{{{{{\rm{H}}}}}}}{{{{{\bf{V}}}}}}{{{{{\boldsymbol{=}}}}}}{{{{{\bf{V}}}}}}{{{{{{\boldsymbol{\Lambda }}}}}}}^{*}$, where ^H^ is the conjugate transpose and ^*^ is the conjugate. The eigenmodes of ${{{{{\bf{L}}}}}}$ capture lagged feedbacks between different climate states that evolve over different time scales. The least damped mode is the mode with the longest decay time, thus representing the slowest varying component—the trend; that is, it is associated with the eigenvalue ${\lambda }_{i}$ with the largest value of $\left|1/{{{{{\rm{Re}}}}}}({\lambda }_{i})\right|$. The spatial pattern (Fig. 2a) of the least damped mode (${{{{{{\bf{u}}}}}}}_{i}$) is obtained as the $i$-th column of ${{{{{\bf{U}}}}}}$, with its time series (gray line in Fig. 2c) obtained by ${{{{{{\bf{v}}}}}}}_{i}^{{{{{{\rm{H}}}}}}}{{{{{\bf{x}}}}}}(t)$, where ${{{{{{\bf{v}}}}}}}_{i}$ is the $i$-th column of ${{{{{\bf{V}}}}}}$. Thus, we identify the trend component, as the projection of ${{{{{{\bf{x}}}}}}}_{{TR}}\left(t\right)={{{{{{\bf{u}}}}}}}_{i}{{{{{{\bf{v}}}}}}}_{i}^{{{{{{\rm{H}}}}}}}{{{{{\bf{x}}}}}}(t)$.

Note that we derive ${{{{{{\bf{x}}}}}}}_{{TR}}\left(t\right)$ from the observed record as well as from each of the CMIP6 realizations. To derive the trend for each CMIP6 realization, we also need to solve the linear operator ${{{{{\bf{L}}}}}}$ separately (from 12/6 PCs of SSTA/SSHA of each CMIP realization). The presented spatial pattern in Figs. 2a and 3a is normalized by its spatial maximum. The presented time series (the gray line in Fig. 2c and the gray line in the bottom panel of Fig. 3c) is normalized by its temporal maximum.

### LIM climate simulations

LIM simulations may be generated by integrating Eq. (1) forward in time, driven by white noise forcing with observationally constrained spatial structure^23^. In addition, the remaining variance (i.e., contained in the unresolved PCs that were not used in LIM construction), denoted as ${{{{{{\bf{x}}}}}}}_{U}$, is approximated as purely white noise,

3 $$\frac{{{{{{\rm{d}}}}}}{{{{{{\bf{x}}}}}}}_{U}}{{{{{{\rm{d}}}}}}t}={{{{{{\boldsymbol{\xi }}}}}}}_{U}$$

where the time-varying white noise is simply approximated by randomly energizing the amplitude of the PCs. See^49^ for details of incorporating the unresolved PCs to construct an untruncated LIM.

In this study, we constructed the LIM by first determining the linear dynamical operator using the observational record of the entire 60-year period. We conducted the eigenanalysis on the dynamical operator, obtained the least damped eigenmode as the LIM trend, and removed the trend component from the observed anomalies. The detrended anomalies were then used to construct the LIM for generating 3000-member ensemble of 60-year-long realizations, representing the detrended dynamical system of 1958–2017 years, i.e., the LIM5817 ensemble. Since the ensemble was generated using a dynamical operator that did not discriminate between the 1958–1987 and 1988–2017 periods, differences between the first and second halves of each 60-year periods can only arise from the system noise, i.e., the ensemble-mean internal variability of the two periods is unchanged.

To represent the full dynamical system, we added the trend component back to each LIM5817 ensemble member, i.e., trend+LIM5817 ensemble. By construction, any statistically significant differences between the two 30-year periods of the trend+LIM5817 ensemble is only due to the externally-forced trend.

We also construct two *new* LIMs to represent each 30-year period, i.e., these LIMs do not see the two periods as having equivalent dynamics. One LIM was constructed using the detrended anomalies of the 1958–1987 period, with the ensemble generated by it therefore providing the detrended realizations of the 1958–1987 period, i.e., LIM5887 ensemble. Similarly, we constructed the other new LIM using the detrended anomalies during 1988–2017, with its generated ensemble denoted as LIM8817 ensemble. The two ensembles represent the detrended dynamical system of each 30-year period, and any possible difference between the two ensembles can also reflect statistically significant changes of the underlying internal variability during the two periods. Note that LIM ensembles of this study are untruncated LIM (see^49^ for details).

### MHW frequency

Frequency was determined by calculating the number of events that exceed ($\ge$) a given intensity for a period longer than ($\ge$) a given duration, divided by the total number of years in the observational record, in units of events per 10 years. The IDF plot was derived from calculating the frequency for each intensity and duration threshold pair, including intensities from 0.1$\sigma$ to 3.1$\sigma$ and durations from 1 to 16 months. For the spatial maps of frequency differences, we first determined the frequency, for each 30 yr period at each location, for MHW events defined by the 2$\sigma$ °C intensity and 1 month duration threshold pair, as in a previous study^1^. Then the frequencies of the two periods were differenced for these global maps.

### Significance tests

Multiple significance tests were carried out in this study, including testing the significance of the mean shifts and the variance changes, and whether the LIM reproduces the observed SST records. In general, the process was as follows: we obtained the observed value and compared that with the range of simulated values provided by the LIM or the CMIP6 ensembles. If the observed value was within (outside) the 95% LIM or CMIP6 ranges, we determined that as insignificant (significant). Details are as follows.

### Significance of mean shifts and variance changes

We obtained the observed mean shifts by subtracting the mean of 1958–1987 from the mean of 1988–2017, in the observational dataset. For each 60-yr LIM5817 ensemble, i.e., alternative 1958–2017 realization, we then computed its simulated mean shift. Since we constructed a 3000-member LIM5817 ensemble, we then have 3000 mean changes at each grid point; that is, 3000 potential changes which can be compared to the observed value. From these 3000 changes, we obtained the 2.5% and the 97.5% values, whose bounds represented the 95% confidence interval. These 95% ranges were compared to the observed mean shifts to determine significance. Results are shown in Fig. 2b and Supplementary Fig. 1.

A similar process was carried out for testing the significance of the variance changes (Fig. 4a). We obtained simulated variance changes by subtracting the variance of the first 30 year from the second in each of the LIM5817 ensemble and compared their 95% ranges to the observed detrended variance changes.

We also repeated these significance tests for CMIP6, including whether the observed mean shifts were within the 95% of the CMIP6 mean shifts, as well as whether the observed variance changes were within the 95% of the CMIP6 variance changes. Results are shown in Figs. 3b and 4b.

### Reproduction of probability distribution

To show that our LIM ensembles realistically reproduce the observed record, we computed the PDFs of each LIM ensemble member and compared them to the observed PDFs. This was first carried out in the trend+LIM5817 ensemble, to validate that the ensemble is a realistic representation of the observed trend. That is, for each 60-yr trend+LIM5817 ensemble member, we computed the PDFs of the simulated 1958–1987 and the PDFs of the simulated 1988–2017. Given the 3000–member trend+LIM5817 ensemble, we have therefore 3000 PDFs for each period. We then obtained the mean, the 2.5% and the 97.5% values of each bin of the PDFs. These are the ensemble mean PDFs and their 95% confidence interval. If the ensemble-mean PDFs overall overlap with the observed PDFs, within the 95% confidence interval, we consider our LIM ensemble able to track the observations. Results are shown in Fig. 2d, and in Supplementary Fig. 2.

A similar process is carried out on the LIM5887 and LIM8817 ensembles, to validate that they realistically capture the internal variability of each 30-yr period. One example is shown by comparing the observed and simulated Kuroshio-Oyashio Extension PDFs (Fig. 4c). Our result shows the ensemble mean PDFs becoming narrower from 1958–1987 to 1988–2017, consistent with the observed PDF changes. Other regions also consistently show observed PDFs captured by these two LIMs (Supplementary Fig. 2).

### Reproduction of MHWs

To show that our LIM ensembles reproduce the MHW statistics of various intensities and durations, we first computed the observed IDF plots of the two 30-yr period and their differences at each grid location. We then derived the simulated IDF plots and their differences from each LIM ensemble member, i.e., resulting in 3000 IDF difference plots at each grid point. These therefore give us the local 2.5% and the 97.5% IDF difference plots. We also focused on a representative pair of thresholds—$2\sigma$ intensity and 1-month duration—and checked whether the observed frequency is within or outside the 95% range of LIM frequency. This was analysed at each grid point, using the trend+(LIM5887, LIM8817) ensemble, with results shown by the gray dots in Fig. 5d, and using the LIM5887 and LIM8817 ensemble, shown in Fig. 6b. We next conducted the analysis on several other pairs of thresholds to show how our results are not sensitive to the choice of the representative thresholds (Supplementary Fig. 7). A similar process was carried out for CMIP6 models, to check whether the observed frequency is within or outside the 95% range of CMIP6 frequency (Figs. 5e and 6c).

## Supplementary information

Supplementary Information

## Data Availability

The observations and reanalyses that support the findings of this study are publicly available. This includes the HadISST^21^ (https://www.metoffice.gov.uk/hadobs/hadisst/), the ERSSTv5^45^ (https://psl.noaa.gov/data/gridded/data.noaa.ersst.v5.html), the COBE^46^ (https://psl.noaa.gov/data/gridded/data.cobe.html), and the ORAS4^47^ (https://www.cen.uni-hamburg.de/en/icdc/data/ocean/easy-init-ocean/ecmwf-ocean-reanalysis-system-4-oras4.html). CMIP6^22^ multi-model realizations are publicly available at https://esgf-node.llnl.gov/search/cmip6/. Large LIM ensembles are available by running the MATLAB code provided at https://github.com/Tongtong-Xu-PSL/LIM or upon request.

## Code availability

All analyses were performed using MATLAB. Codes can be accessed at https://github.com/Tongtong-Xu-PSL/LIM and https://github.com/Tongtong-Xu-PSL/global-MHW.
