# Supplementary material for: An increase in marine heatwaves without significant changes in surface ocean temperature variability
Source: Nat Commun. 2022 Dec 1;13:7396. doi: 10.1038/s41467-022-34934-x (PMC9715661; doi:10.1038/s41467-022-34934-x)
Supplement: Supplementary file 1 — Supplementary Information [file 41467_2022_34934_MOESM1_ESM.pdf]

Supplementary Information

An increase in marine heatwaves without significant changes in surface ocean temperature variability

Tongtong Xu[1,2*], Matthew Newman[1,2], Antonietta Capotondi[1,2], Samantha Stevenson[3], Emanuele Di Lorenzo[4], and Michael A. Alexander[1]

[1]NOAA Physical Sciences Laboratory, Boulder, CO.

[2]CIRES, University of Colorado, Boulder, CO.

[3]Bren School of Environmental Science and Management, University of California, Santa Barbara, CA.

[4]Department of Earth, Environmental, and Planetary Sciences, Brown University.

Contents of this file:

Supplementary Methods

Supplementary Table 1

Supplementary Figures 1-12

Supplementary Methods

Detecting Piecewise Linear Trend

A piecewise linear trend can be represented as follows,

$$\mathrm{s}(t) = \beta_0 + \beta_1 \cdot t + \beta_2 \cdot (t - t_{knot}) \cdot t_{dummy} \tag{S1}$$

where $s(t)$ is a time series of a geographic location, t is time, t_{knot} is the intercept time stamp connecting the earlier trend segment with the latter, t_{dummy} is a dummy variable, such that $t_{dummy} = 0$ if $t \leq t_{knot}$ and $t_{dummy} = 1$ otherwise. β_0, β_1 and β_2 are scalar unknowns to be solved. Representing the piecewise linear trend formation in the vector form, we can solve the trends globally as,

$$\mathbf{s}(t) = \mathbf{A}\,\mathbf{t}, \text{where } \mathbf{t} = \begin{bmatrix} 1 & 1 & \cdots & 1 & 1 & 1 & \cdots & 1 \\ 1 & 2 & \cdots & t_{knot} & t_{knot}+1 & t_{knot}+2 & \cdots & t_N \\ 0 & 0 & \cdots & 0 & 1 & 2 & \cdots & t_N - t_{knot} \end{bmatrix} \tag{S2}$$

and $\mathbf{s}(t) \in R^{S \times N}$ is the spatiotemporal variable (S is the total points in space, and N is the total timestamps). Note that time starts from 1, 2 to t_N, i.e., this vector form works only when the time increment is a constant. Here we take $t_{knot} = 360$. After solving $\mathbf{A} = (\mathbf{s}(t)\mathbf{t}^{\mathrm{T}})(\mathbf{t}\mathbf{t}^{\mathrm{T}})^{-1}$, the trend component is obtained as $\mathbf{s}_{\mathrm{TR}}(t) = \mathbf{At}$.

By removing the piecewise linear trend globally, we reproduce Figs. 2 and 4 as Supplementary Figs. 11-12, for analysis concerning observed record. These analyses serve to additionally demonstrate that (a) our identification of the least damped mode as the trend is robust, (b) our results are not sensitive to the specific detrending methods, and (c) the historical period of 1958-2017 has indeed experienced an intensifying warming over time.

Supplementary Table 1. Time of a historical MHW event reached to a peak intensity.

Acronym	Full name	Time
Beng.	Benguela	04/1995
BoB	Bay of Bengal	12/1987
CCS	California Current System	12/2014
ECS	East China Sea	08/2016
GOA	Gulf of Alaska	01/2014
KOE	Kuroshio-Oyashio Extension	09/2012
Med.	Mediterranean	06/2003
NA	Northern Australia	03/2016
Niño 3.4	-	12/1997
NWA	Northwest Atlantic	05/2012
Peru	-	03/2017
Tas.	Tasman Sea	12/2017
WA	Western Australia	02/2011
WSA	Western South Atlantic	02/2014

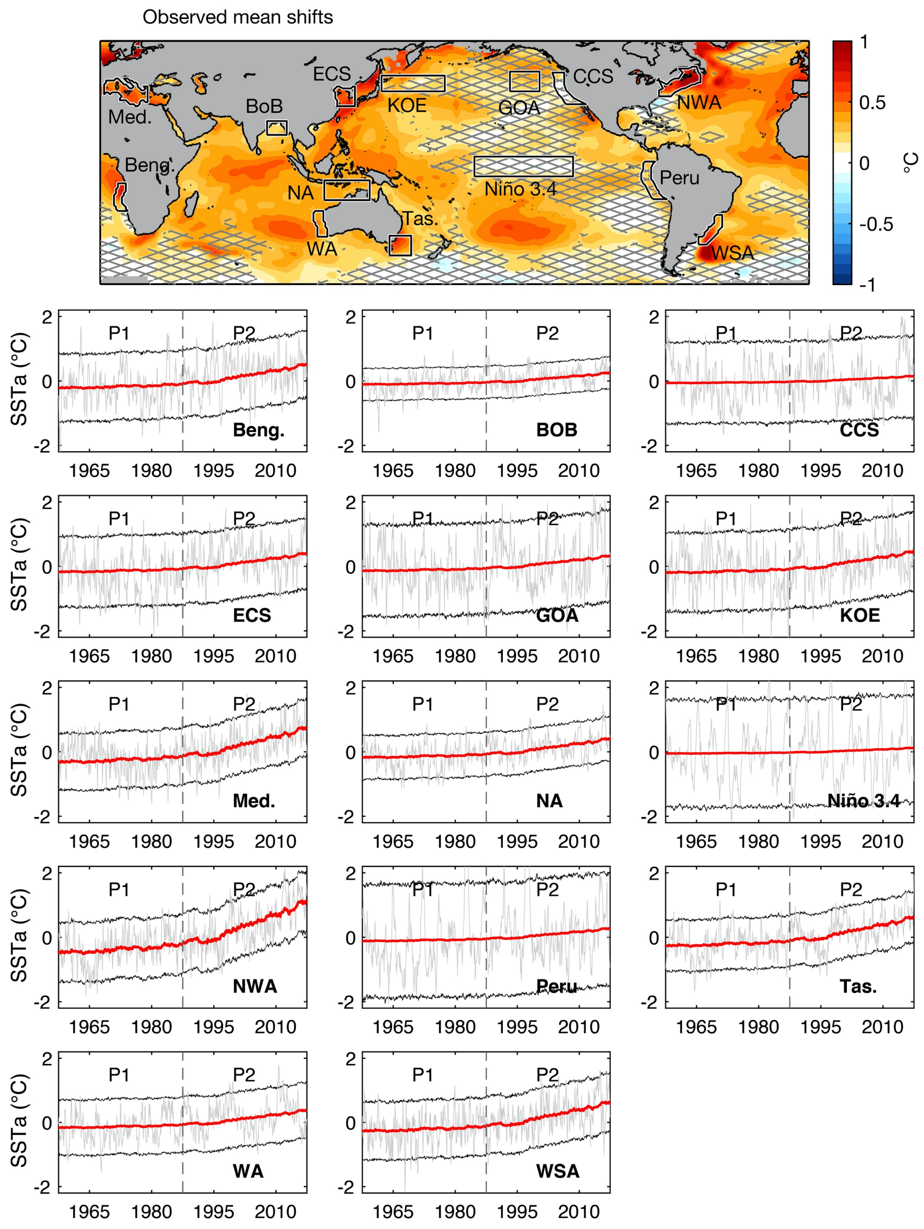

Supplementary Figure 1. Observed mean shifts (shading in the top panel) and their significance (cross-hatched marks where mean shifts are *not significant*); spatially averaged SSTa time series (gray) at targeted regions, along with their trends (red) and 95% confidence intervals (black) from the trend+LIM5817 ensemble. These regional trend lines are identical to Figure 2c. The Northwest Atlantic panel is identical to Figure 1c. Dashed line separates P1 (1958-1987) from P2 (1988-2017).

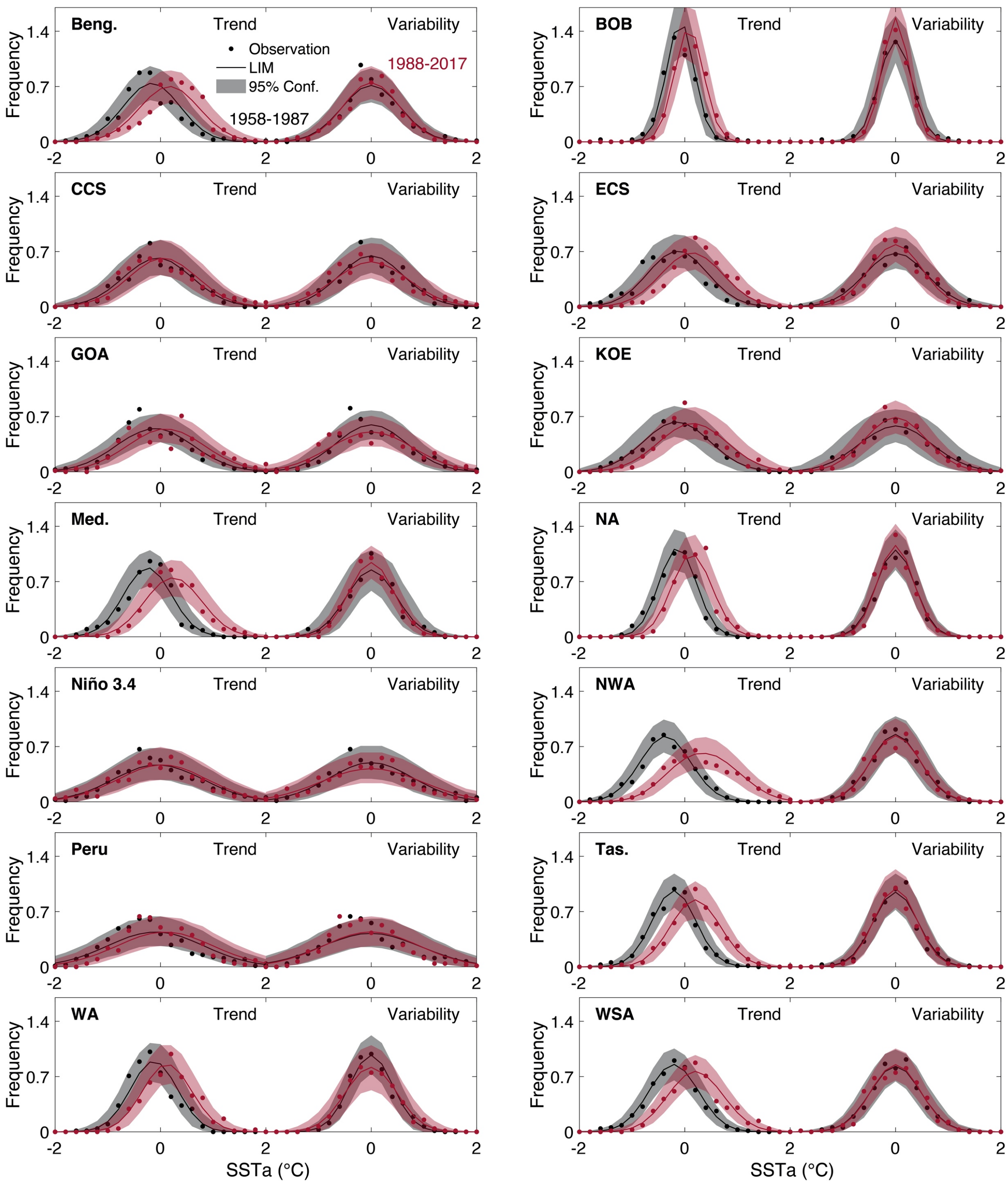

Supplementary Figure 2. 1958-1987 (gray) and 1988-2017 PDF (red) under the impact of SSTa trend (left) vs. variability (right) at targeted regions. Dots are observed PDFs of SSTa (left), detrended SSTa (right). Solid lines are ensemble mean PDFs of trend+LIM5817 (left), LIM5887 and LIM8817 (right). Shadings are 95% confidence interval of those LIM ensembles.

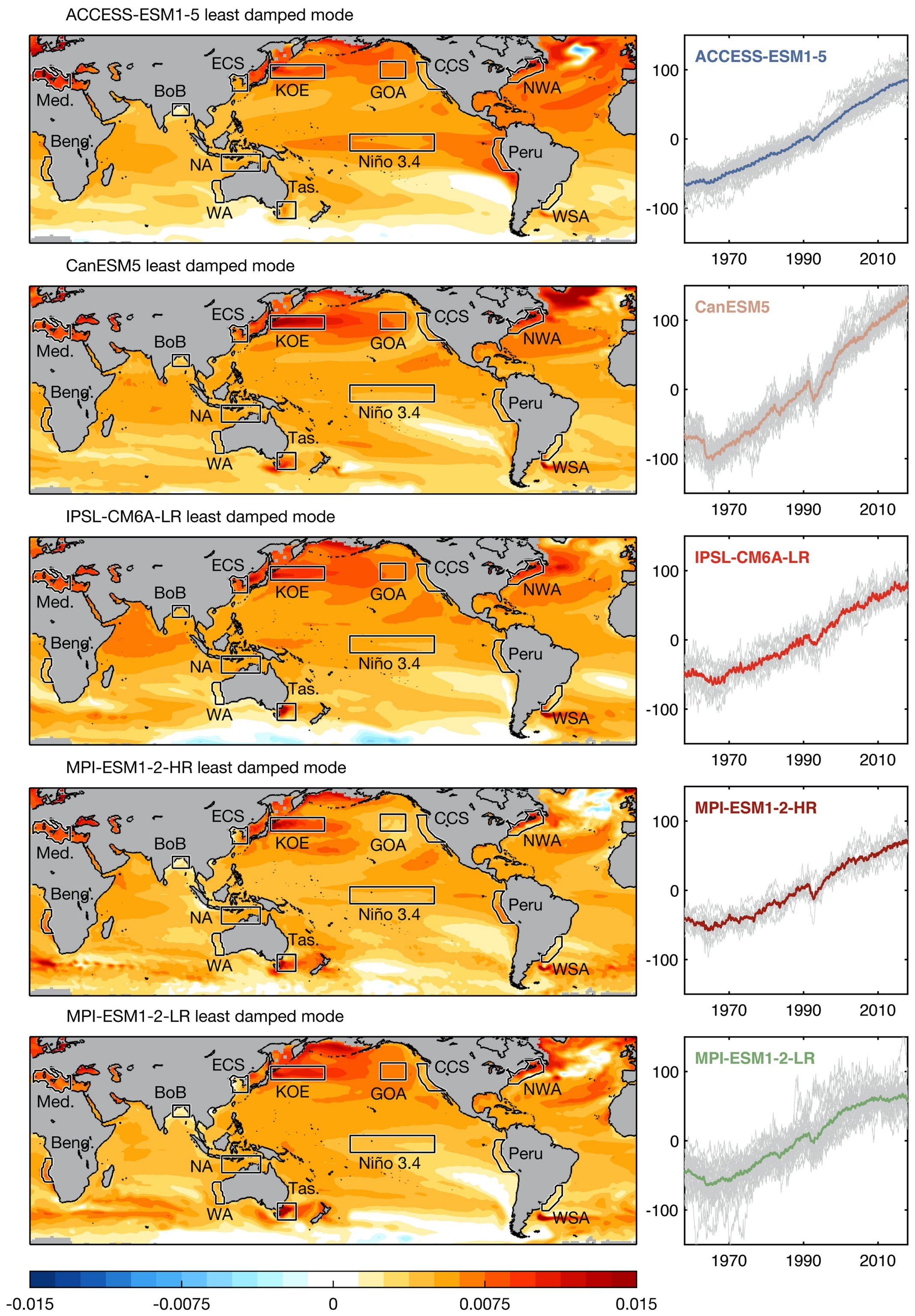

Supplementary Figure 3. Pattern (left) and the time series (right) of the least damped mode, for each CMIP6 model. Left, the mean pattern averaged across all realizations; right, time series of each realization (gray) as well as the mean time series (thick colored line).

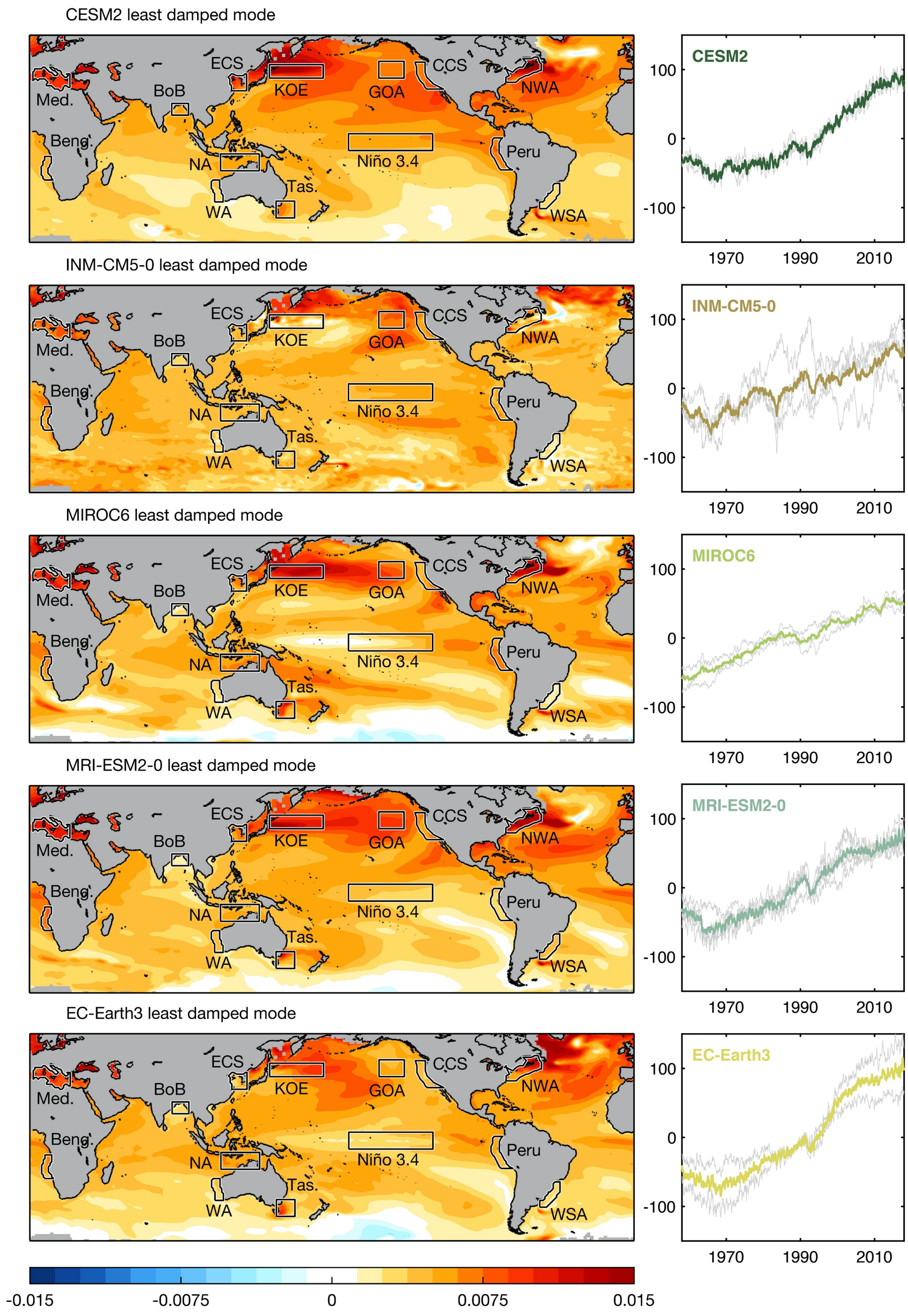

Supplementary Figure 3. (cont.)

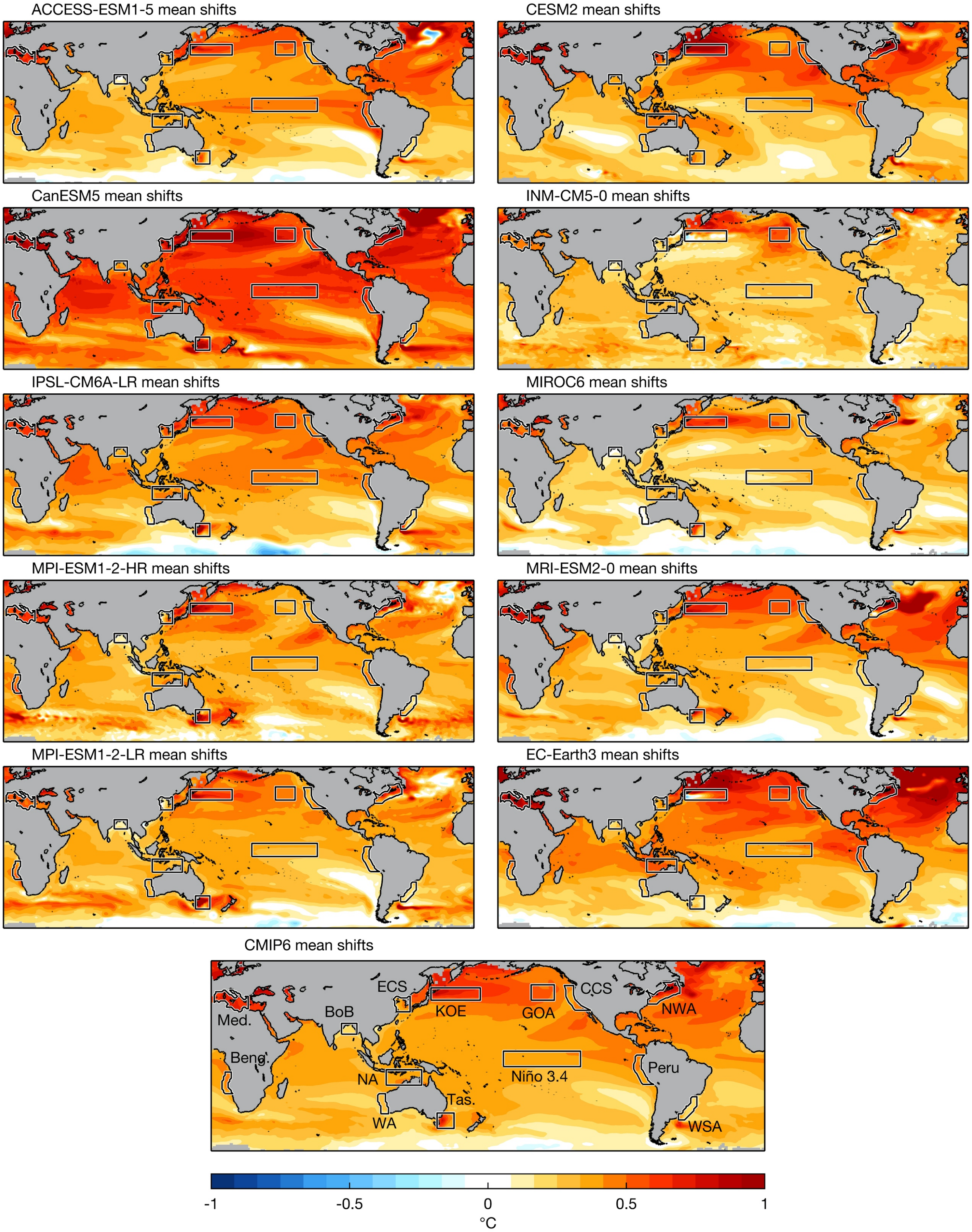

Supplementary Figure 4. Mean shifts, for each CMIP6 model. Top 10 panels, derived by obtaining the mean shifts in each realization of a CMIP6 model and averaging across realizations. Bottom panel, the mean pattern across models, i.e., averaging top 10 panels.

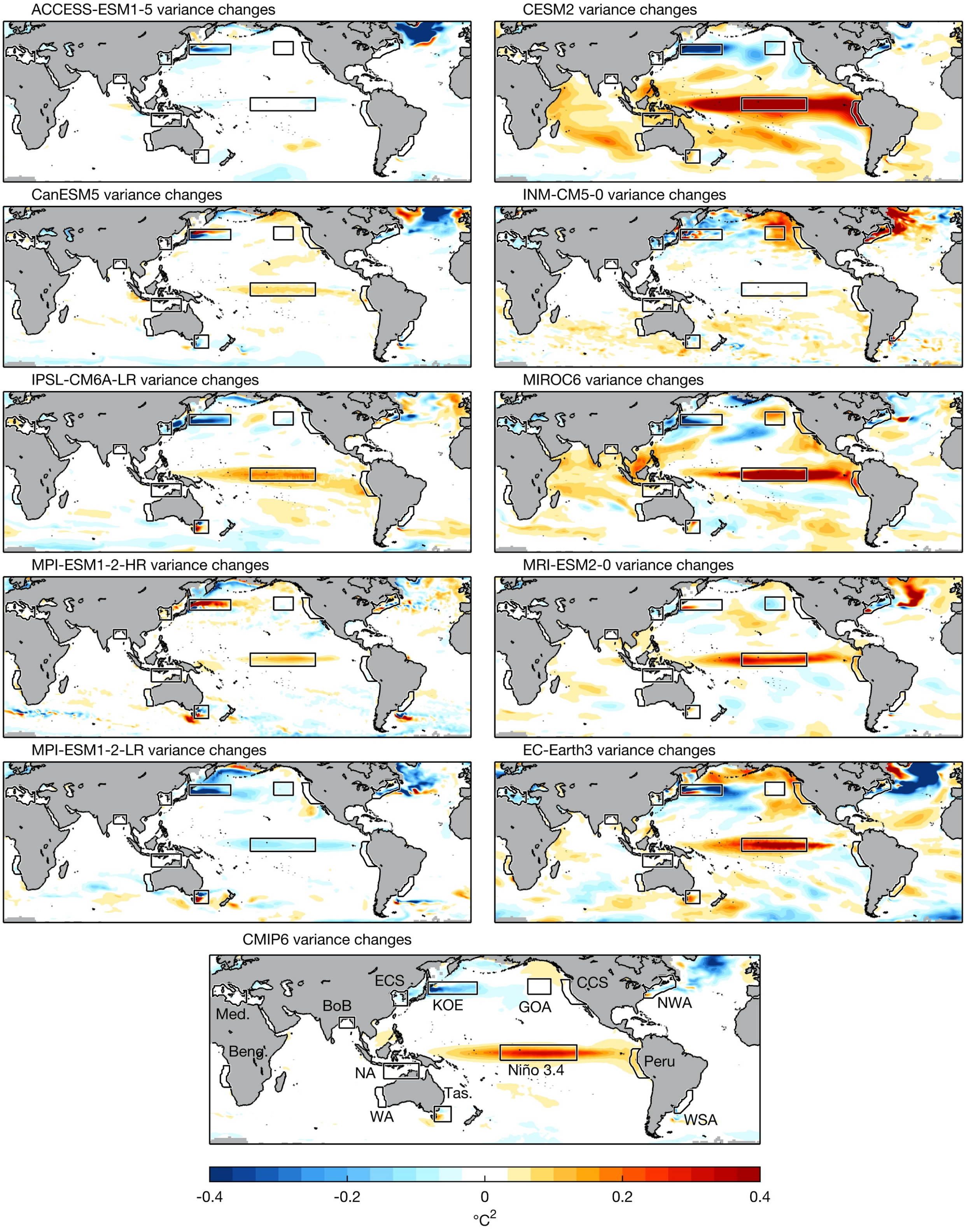

Supplementary Figure 5. Variance changes, for each CMIP6 model. Top 10 panels, derived by obtaining the variance changes in each realization of a CMIP6 model and averaging across realizations. Bottom panel, same as Fig. 4b, obtained by averaging top 10 panels.

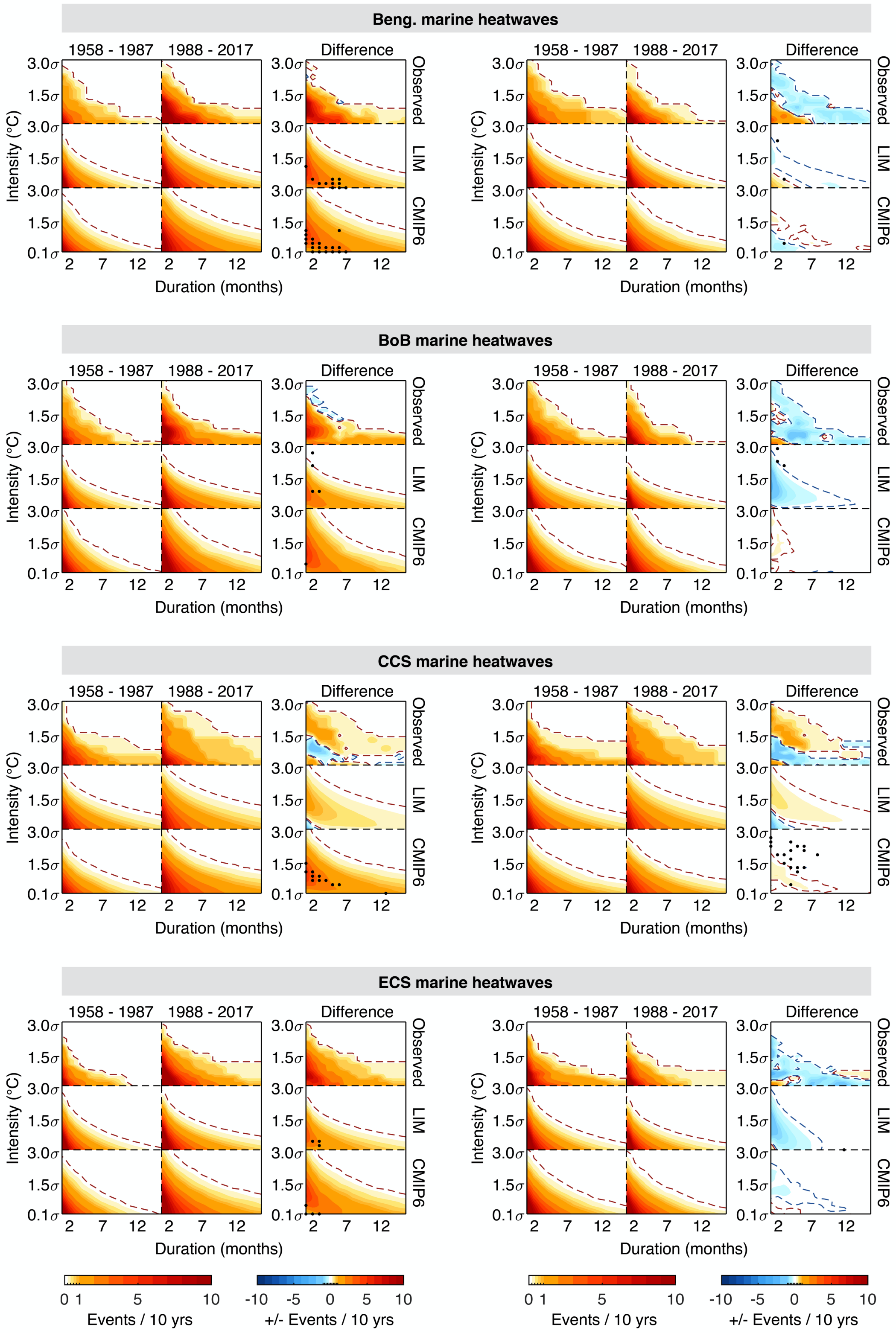

Supplementary Figure 6. IDF plots for each region of interest. Left 3 columns, derived from datasets that include trend; right 3 columns, derived from detrended datasets. For example, the left/right 3 columns of the Northwest Atlantic panel are identical to Fig. 5a/Fig. 5b.

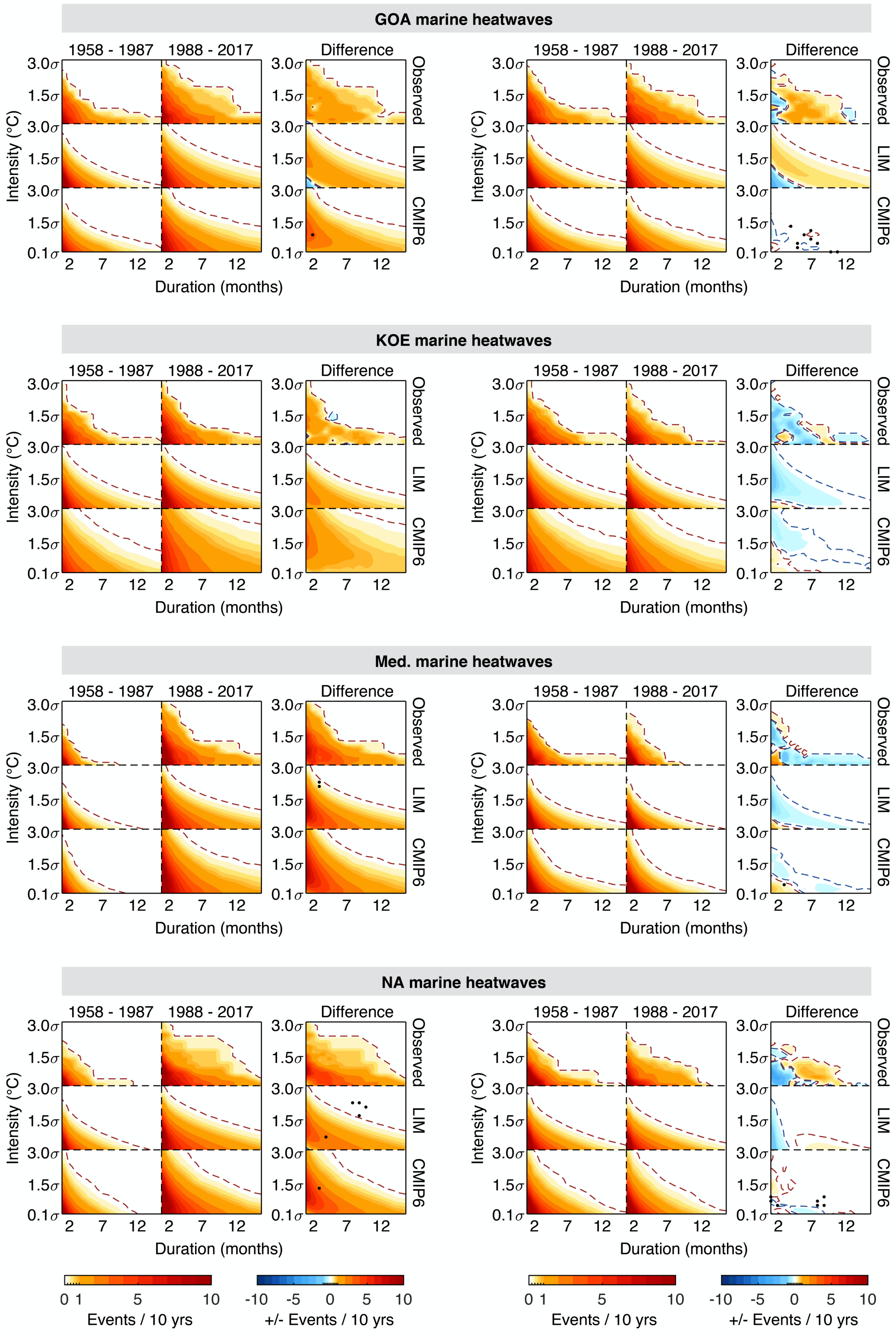

Supplementary Figure 6. (cont.)

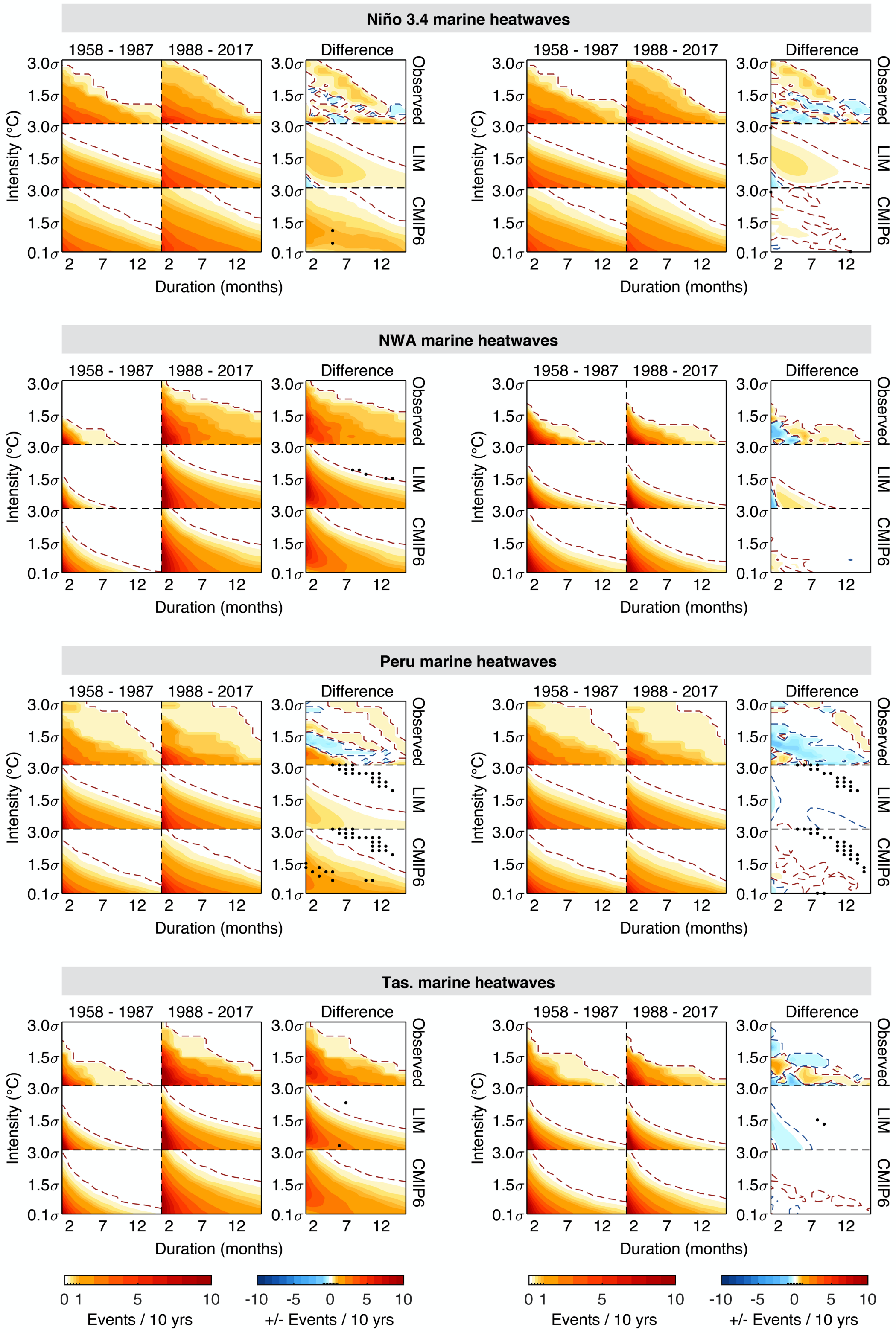

Supplementary Figure 6. (cont.)

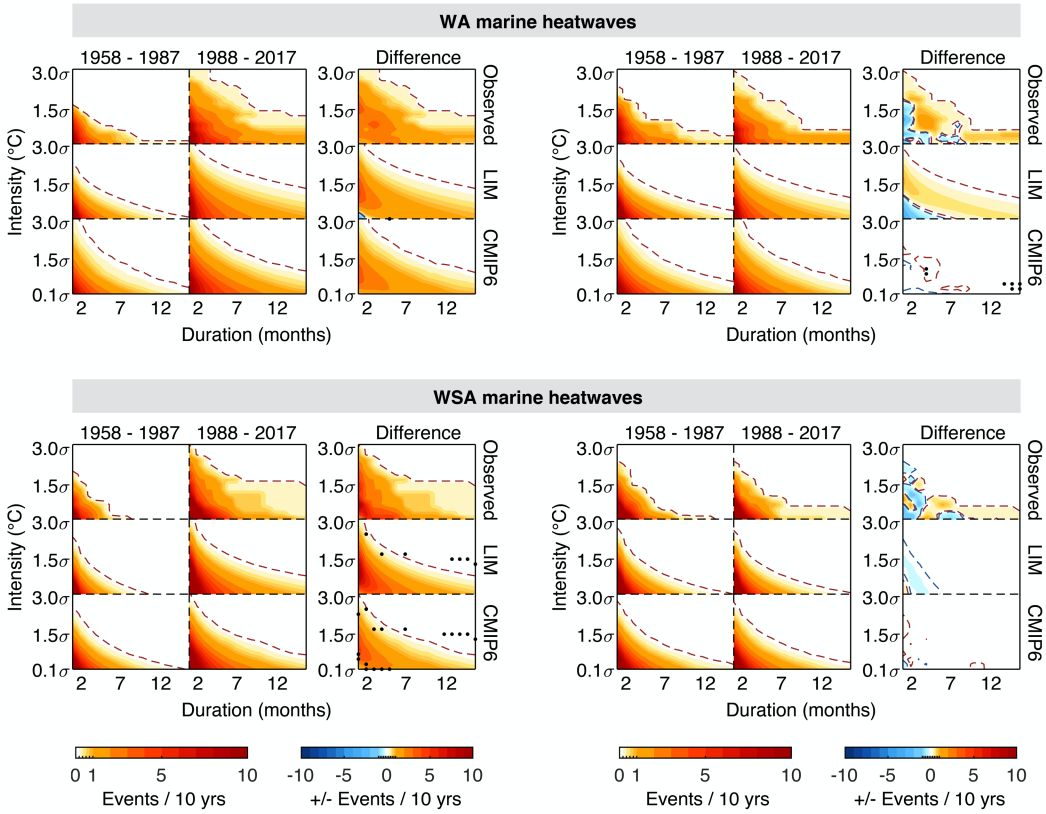

Supplementary Figure 6. (cont.)

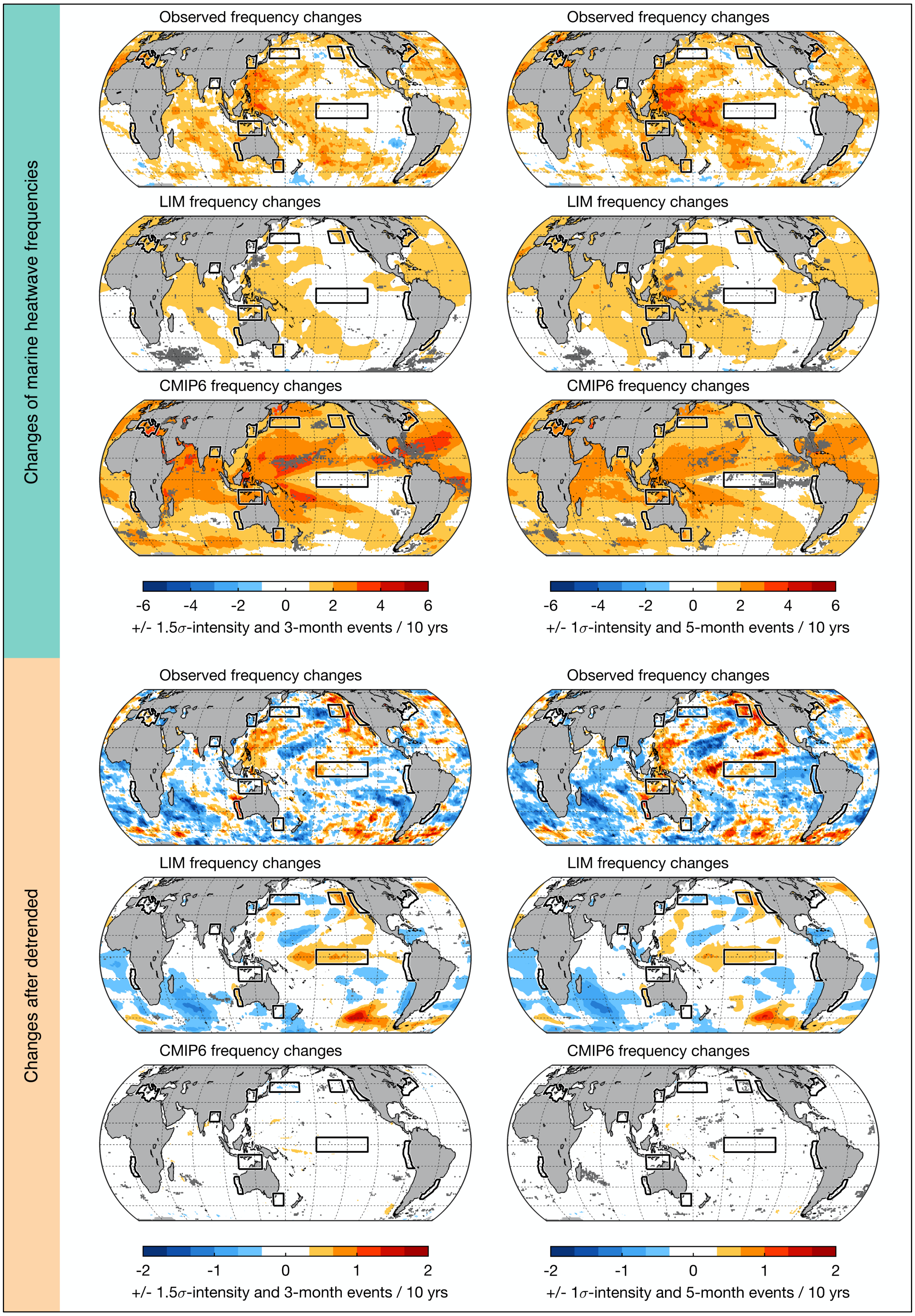

Supplementary Figure 7. Same as Fig. 5c-e, Fig. 6a-c, except for different pairs of intensity and duration thresholds. Threshold pairs are noted next to the colorbar.

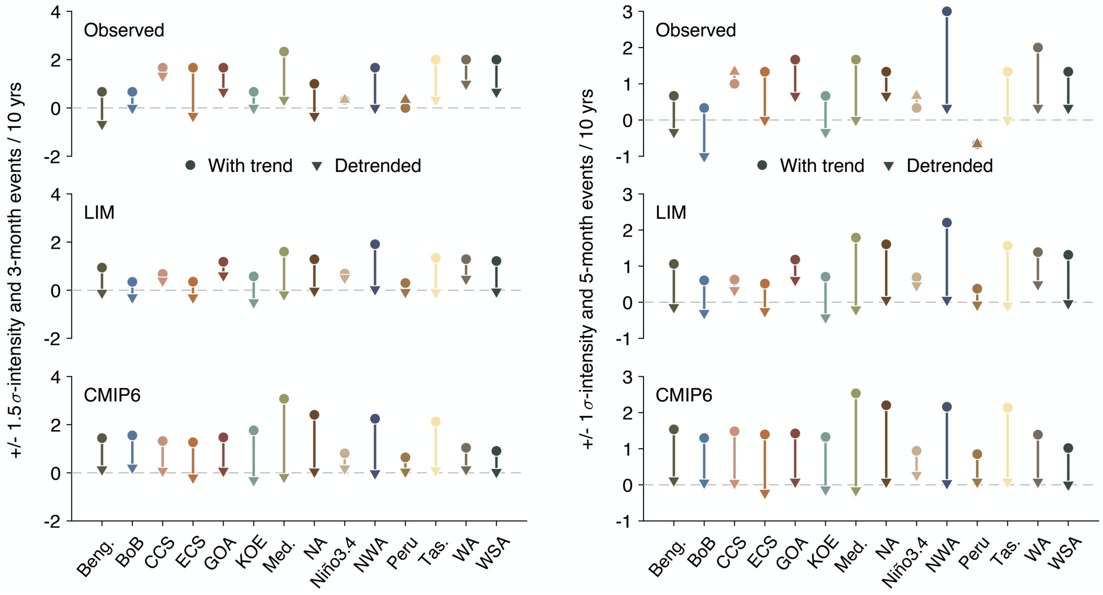

Supplementary Figure 8. Same as Fig. 7, except for different pairs of intensity and duration thresholds. Threshold pairs are noted next to the y-axis.

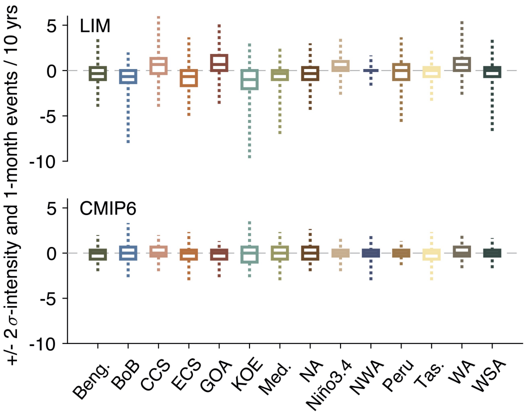

Supplementary Figure 9. Boxplot of changes in MHW occurrences, derived from detrended dataset: (top) LIM and (bottom) CMIP6. We calculate the changes in MHW occurrences between the two periods from each simulation to obtain the range of changes, from which we derive the min, the lower quartile (25%), the median (50%), the upper quartile (75%) and the max.

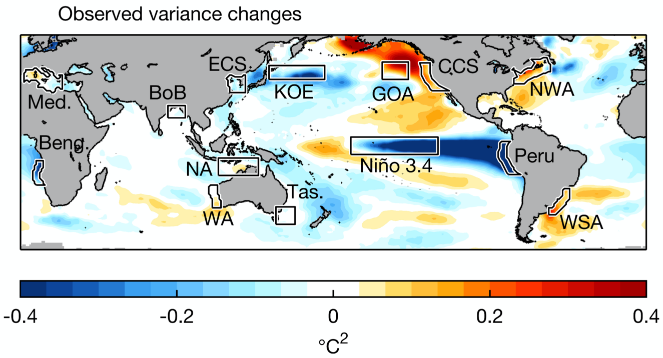

Supplementary Figure 10. Variance changes during post-satellite era, by subtracting variance during 1982-1999 from variance during 2000-2017.

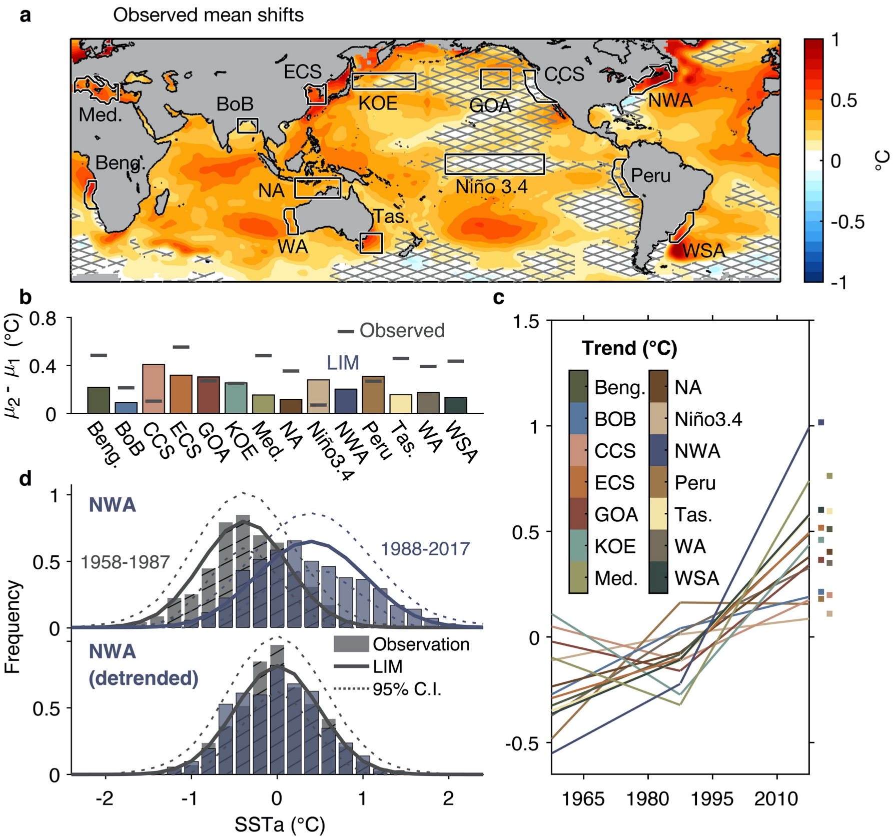

Supplementary Figure 11. Top panel of Supplementary Figure 1 and Figure 2b, c, d reproduced based on piecewise linear trend.

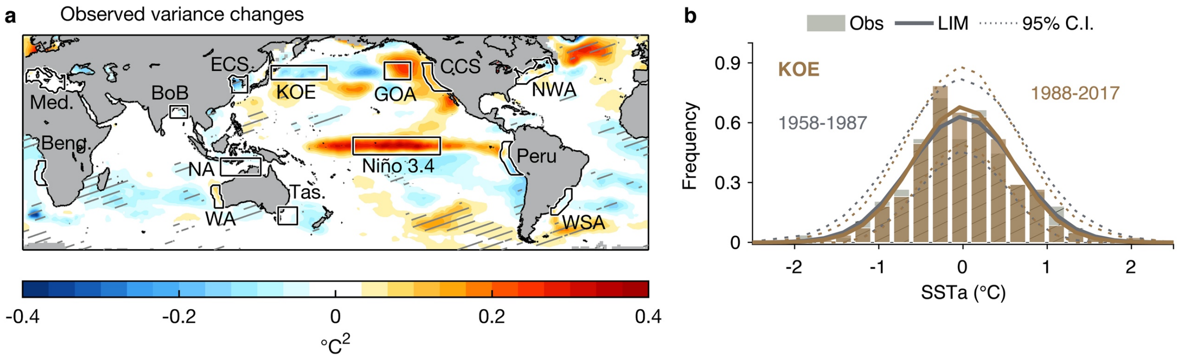

Supplementary Figure 12. Top panels of Figure 4 reproduced based on piecewise linear trend.
